# EANM dosimetry committee recommendations for dosimetry of 177Lu-labelled somatostatin-receptor- and PSMA-targeting ligands

**DOI:** 10.1007/s00259-022-05727-7

**Published:** 2022-03-14

**Authors:** Katarina Sjögreen Gleisner, Nicolas Chouin, Pablo Minguez Gabina, Francesco Cicone, Silvano Gnesin, Caroline Stokke, Mark Konijnenberg, Marta Cremonesi, Frederik A. Verburg, Peter Bernhardt, Uta Eberlein, Jonathan Gear

**Affiliations:** 1 Medical Radiation Physics, Clinical Sciences Lund, Lund University, Lund, Sweden; 2 Université de Nantes, CNRS, Inserm, Oniris, CRCINA, Nantes, France; 3 Department of Medical Physics and Radiation Protection, Gurutzeta-Cruces University Hospital/Biocruces Health Research Institute, Barakaldo, Spain; 4 Department of Applied Physics, Faculty of Engineering, UPV/EHU, Bilbao, Spain; 5 Department of Experimental and Clinical Medicine, Neuroscience Research Centre, PET/RM Unit, “Magna Graecia” University of Catanzaro, Catanzaro, Italy; 6 Nuclear Medicine Unit, University Hospital “Mater Domini, Catanzaro, Italy; 7 Institute of Radiation Physics, Lausanne University Hospital, University of Lausanne, Lausanne, Switzerland; 8 Division of Radiology and Nuclear Medicine, Oslo University Hospital, Oslo, Norway; 9 Department of Physics, University of Oslo, Oslo, Norway; 10 Department of Radiology and Nuclear Medicine, Erasmus Medical Center, Rotterdam, the Netherlands; 11 Department of Medical Imaging, Radboud University Medical Center, Nijmegen, the Netherlands; 12 Radiation Research Unit, Department of Medical Imaging and Radiation Sciences, Istituto Europeo di Oncologia, IRCCS, Milan, Italy; 13 Department of Medical Radiation Sciences, Institute of Clinical Sciences, Sahlgrenska Academy, Gothenburg University, Gothenburg, Sweden; 14 Department of Medical Physics and Biomedical Engineering, Sahlgrenska University Hospital, Gothenburg, Sweden; 15 Department of Nuclear Medicine, University Hospital Würzburg, Würzburg, Germany; 16 Joint department of Physics, Royal Marsden NHSFT and Institute of Cancer Research, Sutton, UK

**Keywords:** Dosimetry, Lutetium-177, Somatostatin-receptor ligands, PSMA-targeting ligands, Neuroendocrine, Prostate adenocarcinoma

## Abstract

The purpose of the EANM Dosimetry Committee is to provide recommendations and guidance to scientists and clinicians on patient-specific dosimetry. Radiopharmaceuticals labelled with lutetium-177 (177Lu) are increasingly used for therapeutic applications, in particular for the treatment of metastatic neuroendocrine tumours using ligands for somatostatin receptors and prostate adenocarcinoma with small-molecule PSMA-targeting ligands. This paper provides an overview of reported dosimetry data for these therapies and summarises current knowledge about radiation-induced side effects on normal tissues and dose-effect relationships for tumours. Dosimetry methods and data are summarised for kidneys, bone marrow, salivary glands, lacrimal glands, pituitary glands, tumours, and the skin in case of radiopharmaceutical extravasation. Where applicable, taking into account the present status of the field and recent evidence in the literature, guidance is provided. The purpose of these recommendations is to encourage the practice of patient-specific dosimetry in therapy with 177Lu-labelled compounds. The proposed methods should be within the scope of centres offering therapy with 177Lu-labelled ligands for somatostatin receptors or small-molecule PSMA.

## Preamble

The European Association of Nuclear Medicine (EANM) is a professional nonprofit medical association that facilitates communication worldwide among individuals pursuing clinical and research excellence in nuclear medicine. The EANM was founded in 1985. These guidelines are intended to assist practitioners in providing appropriate nuclear medicine care for patients. They are not inflexible rules or requirements of practice and are not intended, nor should they be used, to establish a legal standard of care. The ultimate judgement regarding the propriety of any specific procedure or course of action must be made by medical professionals taking into account the unique circumstances of each case. Thus, there is no implication that an approach differing from the guidelines, standing alone, is below the standard of care. On the contrary, a conscientious practitioner may responsibly adopt a course of action different from that set out in the guidelines when, in the reasonable judgement of the practitioner, such course of action is indicated by the condition of the patient, limitations of available resources, or advances in knowledge or technology subsequent to the publication of the guidelines. The practice of medicine involves not only the science but also the art of dealing with the prevention, diagnosis, alleviation, and treatment of disease. The variety and complexity of human conditions make it impossible to always reach the most appropriate diagnosis or to predict with certainty a particular response to treatment. Therefore, it should be recognised that adherence to these guidelines will not ensure an accurate diagnosis or a successful outcome. All that should be expected is that the practitioner will follow a reasonable course of action based on current knowledge, available resources, and the needs of the patient to deliver effective and safe medical care. The sole purpose of these guidelines is to assist practitioners in achieving this objective.

## Background information

### Lutetium-177

The radionuclide lutetium-177 (177Lu) is a rare earth metal that undergoes β^−^ decay to stable hafnium-177 with a half-life of 6.647 days [1]. On decay 177Lu, emits electrons, including β^−^ particles and internal conversion electrons with a mean kinetic energy of 147 keV per decay and maximum electron energy of 497 keV. These energies correspond to ranges (continuous slowing down approximation) in unit-density soft tissue of 0.28 and 1.8 mm, respectively [2]. The decay of 177Lu also results in emission of gamma photons with energies (yields) of 112.9 keV (6.2%), 208.4 keV (10.4%), 249 keV (0.2%), and 321 keV (0.2%), where the two first are useful for patient imaging. Production of 177Lu can be made by two possible routes, either through neutron capture 176Lu(n,γ)177Lu, or indirectly through the reaction 176Yb(n,γ)177Yb→177Lu. In the former route, the long-lived isomer 177mLu is also produced (half-life 160.44 days), forming a low-amount radionuclide impurity mainly of importance for waste management [3, 4].

### 177Lu-labelled somatostatin-receptor ligands

The somatostatin receptor (SSR) is a G-protein coupled transmembrane receptor with the hormone somatostatin as its main ligand. Currently, five distinct subtypes of this receptor have been identified. Derivatives of somatostatin, which bind particularly to SSR subtypes 2 and, to a lesser degree, 5, most notably octreotide and octreotate, have been adapted for radiolabelling to contain the chelator dodecane tetraacetic acid (DOTA). This has resulted in the well-known DOTA-TOC [5] and DOTA-TATE [6] that can be labelled with radionuclides such as 111In, 68Ga, 90Y, or 177Lu. In the further text, the different 177Lu-labelled somatostatin-receptor targeting ligands are collectively referred to as [177Lu]Lu-SSRT.

There is generally a much higher level of SSR expression on neuroendocrine tumour (NET) cells or meningiomas than in normal tissues [7]. The highest accumulation of [177Lu]Lu-SSRT in normal tissues is seen in the liver, the spleen, the kidneys, and the pituitary gland, due to different mechanisms of uptake.

Radionuclide therapy with [177Lu]Lu-DOTA-TATE (Lutathera®) was approved for the treatment of progressive, well-differentiated somatostatin receptor-positive gastroenteropancreatic NETs following the results of phase 3 NETTER-1 trial. The trial randomly assigned 229 patients with well-differentiated metastatic midgut NETs to receive either [177Lu]Lu-DOTA-TATE (7.4 GBq, four infusions every 8 weeks) plus long-acting somatostatin analogues or long-acting somatostatin analogues alone. Twenty-month projected progression-free survival (PFS) was 65.2 vs. 10.8% in the treatment and the control arm, respectively (p < 0.0001). The [177Lu]Lu-DOTA-TATE treatment produced only transient haematological toxicity, with grade 3/4 neutropenia, thrombocytopenia, and lymphopenia occurring in 1%, 2%, and 9% of patients, respectively [8].

### 177Lu-labelled ligands of prostate-specific membrane antigen

Prostate-specific membrane antigen (PSMA), also known as glutamate carboxypeptidase II or folate hydrolase I, is a transmembrane glycoprotein expressed on prostate cells [9, 10]. Small-molecule ligands of PSMA, e.g., PSMA-617 [11, 12] and PSMA imaging and therapy (I&T) [13, 14], have been radiolabelled with 177Lu for the treatment of metastatic prostate adenocarcinoma. In the further text, the different 177Lu-labelled small-molecule PSMA-targeting ligands are collectively referred to as [177Lu]Lu-PSMA.

There is greater PSMA expression in prostate cancer cells than in benign prostate cells, thus providing a relatively specific target for patients with this neoplasm [15]. PSMA is expressed in other tissues besides prostate cancer and benign prostate epithelium, including proximal renal tubules of kidneys, brain, intestine, and in the neovasculature of most solid neoplasms [15, 16]. The highest accumulation of PSMA in normal tissues relevant with regards to [177Lu]Lu-PSMA therapies is in the salivary and lacrimal glands [12, 17–23]. For salivary glands, immuno-histochemistry revealed focal expression of PSMA, and the high uptake of [177Lu]Lu-PSMA is believed to be the result of both specific and non-specific uptake mechanisms [24–26]. Therapy with [177Lu]Lu-PSMA may have profound clinical benefits for some patients, as occasional complete radiological and biochemical responses have been reported [27, 28]. In most patients, however, [177Lu]Lu-PSMA therapy does not result in the full disappearance of disease on imaging [28]. Recently, results of the phase 3 VISION trial were published [29], showing a significant survival benefit for the addition of [177Lu]Lu-PSMA-617 to the standard of care over the standard of care alone in 831 patients with metastatic castration-resistant prostate cancer (median PFS: 8.7 vs. 3.4 months; median overall survival: 15.3 vs. 11.3 months, respectively, both p < 0.001).

## Radiobiological effects on normal tissues and tumours

### Blood elements and bone marrow

As [177Lu]Lu-SSRT and [177Lu]Lu-PSMA are administered intravenously, the blood elements are the first to be exposed to radiation. The major determinant of radiation exposure of the haematopoietic stem cells is radiopharmaceutical circulation within the bone marrow. However, specific targeting mechanisms to more differentiated blood cell progenitors may also contribute. For instance, SSRs are overexpressed on activated leucocyte subtypes, such as lymphocytes and monocytes [30]. Additional factors affecting haematologic toxicity are the extent of bone metastatic involvement and previous history of myelotoxic chemotherapy or bone marrow irradiation. Haematologic toxicity is the most common adverse event after 177Lu therapy. Grade 3–4 toxicity, most often thrombocytopenia, has been observed in 10–15% of patients treated with [177Lu]Lu-SSRT [7, 31–33] and in approximately 10% of those treated with [177Lu]Lu-PSMA [34]. The occurrence of secondary myelodysplastic syndrome or acute leukaemia has been observed several years after treatment with [177Lu]Lu-SSRT [33, 35, 36].

Weak but significant correlations between image-based estimates of the red-marrow absorbed dose and haematological toxicity have been demonstrated [37–40]. Moreover, elevated levels of DNA damage in peripheral blood lymphocytes have been identified using biomarkers such as γ-H2AX and 53BP1 [41–44]. The threshold bone-marrow absorbed dose for severe haematologic toxicity is generally considered to be 2 Gy, in analogy to the experience with 131I therapy [45], but confirmation of this 2 Gy threshold is still needed for applications with 177Lu-based therapies. Interestingly, in a phase I trial for therapy with the SSRT-antagonist [177Lu]Lu-satoreotide tetraxetan, patients (3/20) with a bone marrow absorbed dose above 1.5 Gy developed grade 4 thrombocytopenia [46].

### Kidneys and liver

Abdominal organs are irradiated in [177Lu]Lu-SSRT and [177Lu]Lu-PSMA therapies due to radiopharmaceutical-specific uptake or their physiological excretory functions. The kidney is generally considered the dose-limiting organ in therapy with [177Lu]Lu-SSRT, owing to unspecific uptake mechanisms by proximal tubular cells [47]. Acute radiation nephropathy manifests between 6 months and 1 year after irradiation with typical signs of renal failure, including proteinuria, anaemia, hypertension, and congestive heart failure. Chronic radiation-induced nephropathy consists of vascular damage in combination with progressive loss of parenchymal cells. This may follow the acute syndrome or present years after irradiation [48–51]. To reduce the risk of renal toxicity after administration of [177Lu]Lu-SSRT, protocols for renal protection have been developed involving co-infusion of amino acids that compete for the megalin receptor on tubular cells. Immediate and reversible side effects following therapy, like vomiting and cramps, are ascribed to renal-protection protocols rather than radiation exposure [52, 53]. For [177Lu]Lu-SSRT, including concurrent kidney protection, the level of reported nephrotoxicity is limited to disease-related events, and for therapy with [177Lu]Lu-PSMA, it appears to be negligible at current activities [28, 54]. This indicates that the tolerance absorbed doses for kidneys exceed those given so far, possibly owing to nonuniform irradiation and modest absorbed dose rates. With the ambition to increase the treatment efficacy, dosimetry-guided clinical trials for therapy of NETs with [177Lu]Lu-SSRT have been undertaken [32, 40, 55] applying renal absorbed dose or biologically effective dose (BED) constraints extrapolated from external-beam radiotherapy (EBRT) of either 23 Gy or 28 Gy [51, 56], or 40 Gy for patients without risk factors [57]. In therapy using [90Y]Y-SSRT, a BED-dependent annual creatine clearance loss was identified [58], and retrospective data analysis indicated a BED limit of approximately 39 Gy for a 5% incidence [57, 59].

The liver is generally not considered an organ at risk for [177Lu]Lu-SSRT or [177Lu]Lu-PSMA therapies, and the liver function has been shown to improve after [177Lu]Lu-SSRT therapy [60]. However, the liver needs to be monitored in case of concomitant treatments and for therapy with larger molecules such as 177Lu-labelled monoclonal antibodies [61]. Classic radiation-induced liver disease develops a few weeks after irradiation and shows the typical pathologic appearance of veno-occlusive disease of the central lobule and the small branches of the hepatic veins [62].

### Salivary, lacrimal, and pituitary glands

The major salivary glands comprise three pairs of glands, the parotid, submandibular, and sublingual glands. Recently, a fourth pair of salivary glands were identified after analysing [68Ga]Ga-PSMA PET imaging, the tubarial glands in the nasopharynx region [63]. Radiation exposure may cause xerostomia, a reduction of salivary flow in the oral cavity. Xerostomia is a documented side effect in patients given [177Lu]Lu-PSMA [28, 54], although the tolerance absorbed dose for salivary glands has not yet been identified. Experience from EBRT indicates a low incidence of toxicity below a mean absorbed dose to both parotid glands of approximately 10 Gy, and an absorbed dose limit of 20 Gy has been proposed [64]. Methods for the protection of salivary glands, such as the administration of folic polyglutamate tablets or cooling with icepacks, are being evaluated clinically [65].

The lacrimal glands are paired exocrine glands in the upper lateral region of the two eye orbits. [177Lu]Lu-PSMA exhibits accumulation in the lacrimal glands [66], which have been identified as possibly dose-limiting [67], although no significant occurrence of xerophthalmia (dry eyes) has been reported so far. Xerophthalmia has occasionally been shown to be the dose-limiting toxicity after [225Ac]Ac-PSMA-617 therapy at the highest administered activities [26]. In EBRT, an absorbed dose constraint for the lacrimal glands of 25 Gy was indicated [68].

The pituitary gland, or hypophysis, is located at the base of the brain in a skeletal hollow termed sella turcica (“Turkish saddle”). It has a high expression of SSRs and is thus targeted by [177Lu]Lu-SSRT. Radiation exposure may affect the hypothalamic-pituitary axis, a key regulator of endocrine function. Different hormone-secreting cell types have different radiosensitivity, with somatotropic and thyrotropic cells being the most and least radiosensitive pituitary cells, respectively. In EBRT, an absorbed dose limit of 20 Gy is recommended to avoid growth hormone (GH) deficiency. The absorbed dose limit for panhypopituitarism is 45 Gy [68]. Complex feedback loops compensate for hormonal variations, which make it challenging to assess short-term mild endocrine toxicities. The few available studies on pituitary function following [177Lu]Lu-SSRT suggest the occurrence of mild chronic impairment of the GH/IGF-1 and gonadotropin axes after repeated treatment cycles [69, 70]. A statistically significant decrease in the IGF-1 levels was observed, which correlated with both the number of given cycles and the estimated absorbed dose to the pituitary gland [69].

### Tumours

In [177Lu]Lu-SSRT therapy of NETs current evidence points at the existence of relationships between the absorbed dose and response, although data are yet limited and the target absorbed dose for an effective treatment is to be defined. A tumour-volume reduction was observed in the therapy of mixed NETs using 86Y-based dosimetry for [90Y]Y-DOTA-TOC therapy [71]. For [177Lu]Lu-DOTA-TATE, relationships of the diameter- or volume-reduction and their association with the cumulative absorbed dose evaluated at the time of best response were presented for both pancreatic NET and small-intestinal NETs [72, 73]. For [177Lu]Lu-PSMA, there were observations of a significantly higher absorbed dose for PSA-responders (median of 14 Gy) versus nonresponders (median < 10 Gy) when the mean absorbed dose was calculated across all metastases [74].

## Absorbed dose calculation for 177Lu

Following the medical internal radiation dose (MIRD) formalism [75–78], the mean absorbed dose rate $\dot{D}\left({r}_{T},t\right)$ to a target region ${r}_{T}$ from the activity $A\left({r}_{S},t\right)$ located in a source region ${r}_{S}$ at time $t$ after radiopharmaceutical administration is given by

1 $$\dot{D}\left({r}_{T},t\right)= \sum_{{r}_{S}}A\left({r}_{S},t\right) S\left({r}_{T} \leftarrow {r}_{S},t\right).$$

The $S$-value, $S\left({r}_{T}\leftarrow {r}_{S},t\right)$, describes the mean absorbed dose rate at time $t$ delivered to ${r}_{T}$ per unit of activity in ${r}_{S}$. Usually, the time-independent $S$-value, $S\left({r}_{T}\leftarrow {r}_{S}\right),$ is assumed. $S\left({r}_{T}\leftarrow {r}_{S}\right)$ is derived from basic physical principles following

2 $$S\left({r}_{T} \leftarrow {r}_{S}\right)= \frac{1}{m\left({r}_{T}\right)} \sum_{i}{E}_{i} {Y}_{i} \phi \left({r}_{T} \leftarrow {r}_{S},{E}_{i}\right) ,$$

where $m\left({r}_{T}\right)$ is the target region mass, and $\phi$ is the absorbed fraction (AF), i.e., the fraction of the energy emitted from ${r}_{S}$ that is absorbed in ${r}_{T}$. The radionuclide-specific factors ${E}_{i}$ and ${Y}_{i}$ represent the mean energy emitted in a given nuclear transition and the corresponding yield. For 177Lu, the mean energy emitted per decay can be grouped into ${\Delta }_{177\mathrm{Lu},\mathrm{ph}}$ for photon emissions (gamma-photons and X-rays) and ${\Delta }_{177\mathrm{Lu},\mathrm{e}}$ for electron emissions (${\beta }^{-}$ particles, conversion, and Auger electrons). Values of ${\Delta }_{177\mathrm{Lu},\mathrm{ph}}$ and ${\Delta }_{177\mathrm{Lu},\mathrm{e}}$ based on different radionuclide data sets and used in different dosimetry software are summarised in Table 1.

**Table 1:** Emitted energy per 177Lu decay from photon (${\Delta }_{177\mathrm{Lu},\mathrm{ph}}$) and electron (${\Delta }_{177\mathrm{Lu},\mathrm{e}}$) emissions

Use	${{\varvec{\Delta}}}_{177\mathbf{L}\mathbf{u},\mathbf{p}\mathbf{h}}$	${{\varvec{\Delta}}}_{177\mathbf{L}\mathbf{u},\mathbf{e}}$	Reference
Olinda v.1 and v.2 [79]	35.1 keV Bq^−1^ s^−1^ 0.02024 mJ MBq^−1^ h^−1^	147.2 keV Bq^−1^ s^−1^ 0.08490 mJ MBq^−1^ h^−1^	HPS, Stabin, da Luz [80] #
IDAC-Dose 2.1 and OpenDose [81, 82]	35.1 keV Bq^−1^ s^−1^ 0.02024 mJ MBq^−1^ h^−1^	147.9 keV Bq^−1^ s^−1^ 0.08532 mJ MBq^−1^ h^−1^	ICRP 107 [83]
National Nuclear data center, NuDat2 ##	33.4 keV Bq^−1^ s^−1^ 0.01927 mJ MBq^−1^ h^−1^	147.1 keV Bq^−1^ s^−1^ 0.08484 mJ MBq^−1^ h^−1^	Kondev [1]

^#^Health Physics Society (HPS) http://hps.org/publicinformation/radardecaydata.cfm

^##^ www.nndc.bnl.gov/nudat2/

It is noted that ${\Delta }_{177\mathrm{Lu},\mathrm{e}}$ is 4.2–4.4 times higher than ${\Delta }_{177\mathrm{Lu},\mathrm{ph}}$. It is also seen that although different data sets are similar, they are not identical: ${\Delta }_{177\mathrm{Lu},\mathrm{e}}$ is 0.5% higher for ICRP 107 compared to more recent NuDat2 data, and ${\Delta }_{177\mathrm{Lu},\mathrm{ph}}$ from both HPS and ICRP 107 are 5% higher than for NuDat2. For dosimetry, the important point is to be aware that different sets of $S$-values are based on different sets of radionuclide data.

From Eqs. 1 and 2, the mean absorbed dose to a target region is calculated by integration over time:

3 $$D \left({r}_{T}, \tau \right)={\int }_{0}^{\tau }\dot{D}\left({r}_{T},t\right)\mathrm{ d}t =\sum_{{r}_{s}}\tilde{A }\left({r}_{S}, \tau \right)S\left({r}_{T}\leftarrow {r}_{S}\right) ,$$

where the dose-integration period $\tau$ is usually taken as infinity. The time-integrated activity (TIA), $\tilde{A }\left({r}_{S}, \infty \right),$ then represents the total number of radioactive decays that occur in a source region. The TIA is calculated from the time integral of the time-activity curve (TAC) for the source region, where the TAC is derived from a time-sequence of activity measurements. $S$-values are determined analytically or by Monte-Carlo simulations for each radionuclide and source-target-region combination. Such data have been made available for anatomical geometries, including organs and spheres, with uniform distributions of activity and mass density [79, 81, 82, 84]. Generally, the absorbed dose can be considered a sum of self-absorbed dose (when ${r}_{T}={r}_{S}$, i.e. the absorbed dose delivered by activity residing in the target region itself) and cross-absorbed dose (when ${r}_{T}\ne {r}_{S},$ the absorbed dose contribution from activity located in other source regions). When the range of particle emissions is much shorter than the organ dimensions, the AF for self-absorbed dose is very near or equal to unity [85]. This observation forms the basis for the estimation of patient-adjusted $S$-values (${S}_{\mathrm{pat}})$, by scaling of the reference-model $S$-values (${S}_{\mathrm{ref}})$ by the ratio of the reference-model mass ${m}_{\mathrm{ref}}\left({r}_{T}\right)$ to the patient organ mass ${m}_{\mathrm{pat}}\left({r}_{T}\right)$, following

4 $${S}_{\mathrm{pat}}\left({r}_{T}{\leftarrow r}_{S}\right)\approx \frac{{m}_{\mathrm{ref}}\left({r}_{T}\right)}{{m}_{\mathrm{pat}}\left({r}_{T}\right)}\cdot {S}_{\mathrm{ref}}\left({r}_{T}{\leftarrow r}_{S}\right) ; \left({r}_{T}={r}_{S}\right).$$

Equation 4 relies on the assumption that the difference between the photon AF:s for the two masses does not contribute to a significant error in ${S}_{\mathrm{pat}}$. More elaborate methods for the estimation of the photon absorbed fractions have been presented [86, 87].

For source regions with high activity accumulation and retention, located in a surrounding with modest activity, the self-absorbed dose is generally the dominant contributor for 177Lu. The self-absorbed dose is, in turn, dominated by the electron emissions, since: (i) ${{\Delta }_{177\mathrm{Lu},\mathrm{e}}>\Delta }_{177\mathrm{Lu},\mathrm{ph}}$, (ii) the electrons have short ranges in soft tissue and bone, and (iii) the self-absorbed fractions for the photons are low for objects with dimensions typical for many organs [86]. The approximation of electron local-energy deposition (LED) is based on the assumption that when ${r}_{T}={r}_{S}$ (Eq. 2), the AF is equal to unity for electrons and zero for photons, thus giving ${S}_{177\mathrm{Lu}}\left({r}_{T} \leftarrow {r}_{S}\right)\approx {\Delta }_{177\mathrm{Lu},\mathrm{e}}/m\left({r}_{T}\right)$. Table 2 shows the self-absorbed energy per unit of TIA for 177Lu, calculated as the product of the mass and the self-dose $S$-value, based on reference-model data from one example software [81]. Table 2 also shows the error introduced if only considering LED.

**Table 2:** Self-absorbed energy per unit of TIA based on $S$-values for 177Lu for the adult male phantom from IDAC-Dose 2.1 [81], and on the approximation of LED using ${\Delta }_{177\mathrm{Lu},\mathrm{e}}$ from ICRP 107 (Table 1)

$S$-value for self-absorbed dose	Mass (g)	$S$-value (mGy MBq^−1^ h^−1^)	Mass × $S$-value (mJ MBq^−1^ h^−1^)	Ratio to ${\Delta }_{177\mathrm{Lu},\mathrm{e}}$
$S(\mathrm{kidney }\leftarrow \mathrm{kidney})$	422	0.204	0.0861	1.01
$S(\mathrm{liver }\leftarrow \mathrm{liver})$	2360	0.0376	0.0887	1.04
$S(\mathrm{spleen }\leftarrow \mathrm{spleen})$	228.4	0.377	0.0861	1.01
$S(\mathrm{red marrow}\leftarrow \mathrm{red marrow})$	1394	0.0349	0.0487	0.57
$S(\mathrm{blood }\leftarrow \mathrm{blood})$	1	85.3 (in 1 mL)	0.0853	1
LED:${\Delta }_{177\mathrm{Lu},\mathrm{e}}$	N/A	N/A	0.0853	1

**Fig. 1:**
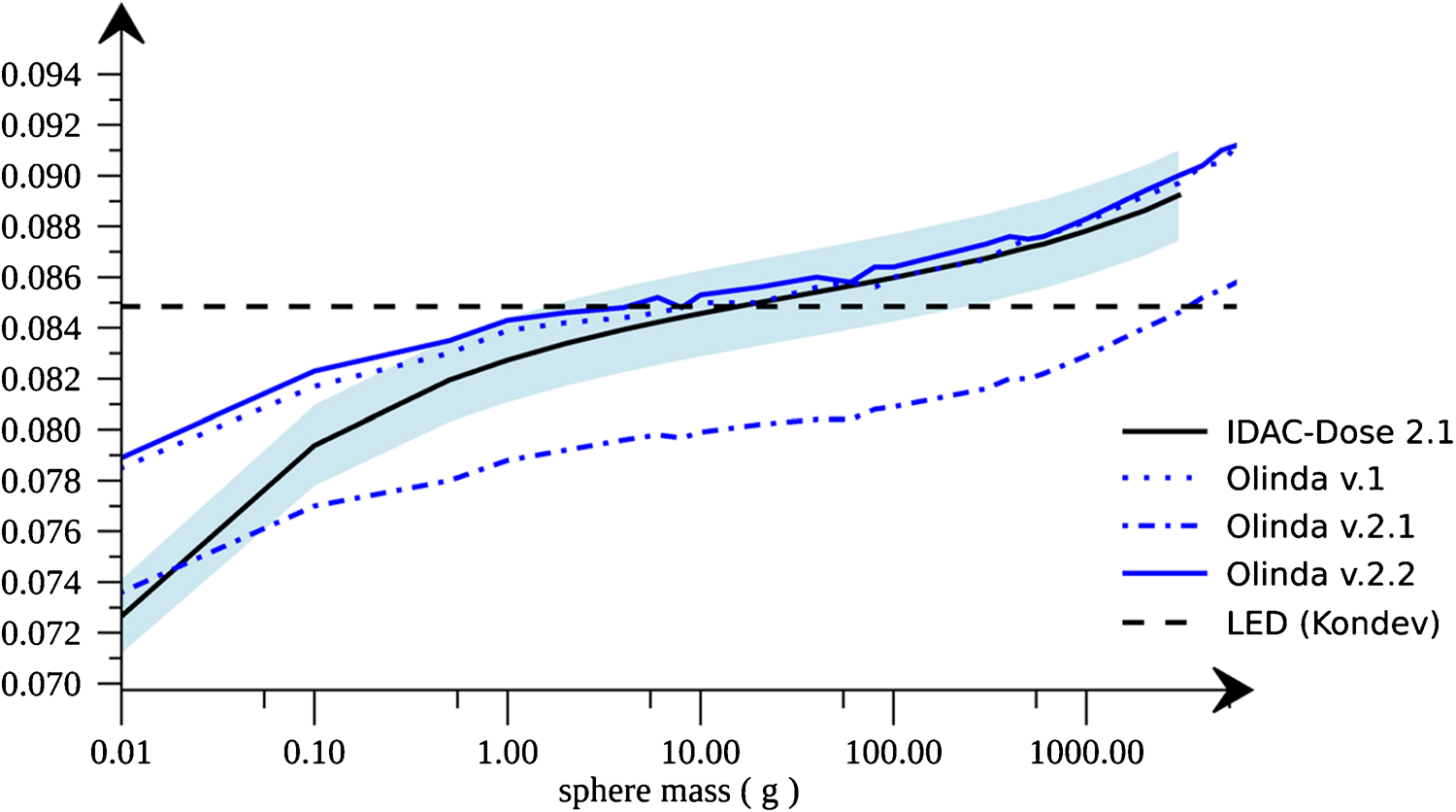
Self-absorbed energy per unit of TIA for 177Lu as a function of mass, based on $S$-values for unit density spheres for IDAC-Dose 2.1, Olinda v.1, v.2.1, and v.2.2. The result of LED from recent radionuclide data is also shown (dashed horizontal line) [1]. The blue band indicates an offset of ±2% from the values for IDAC-Dose 2.1

As noted, for the kidney and spleen, the product mass × $S$ is only 1% higher than that based on LED. These source regions have convex shapes, while for a complex source region such as the red marrow (with a high surface-to-volume ratio), the LED digresses from that calculated from $S$-values. The influence of the size of source/target regions is further illustrated in Figure 1.

As noted, the self-absorbed energy from Olinda v.1, Olinda v.2.2, and IDAC-Dose 2.1 are consistent, and for target regions with a mass between 2 and 300 g, the values agree to within 2%. The values for Olinda v.2.1 are for unknown reasons lower and inconsistent with Olinda v.1 and v.2.2. While the importance of the photon contribution increases as the dimensions of the source/target region increase, there is for smaller regions an increasing escape of electron energy. Both effects cause a difference with respect to the LED value. However, for source/target regions with comparably convex shapes and mass between approximately 2 and 300 g (e.g., kidneys, spleen, salivary glands, and many tumours), the self-absorbed dose calculated by LED is within 1–2% from that using $S$-values. Such small deviations are of the same order of magnitude as those between different sets of radionuclide or $S$-value data.

In principle, the MIRD formalism is not limited to a specific geometry. When the source is located in a point in a uniform medium and the deposited energy scored in symmetric shells around the source, the $S$-value distribution is generally termed a dose-point kernel (DPK) [88]. Likewise, the source can be uniformly distributed in a central voxel and the energy scored in surrounding voxels to produce voxel $S$-values (VSV) [89, 90]. For voxel-based dosimetry, the DPK or VSV is convolved with the activity distribution from a quantitative SPECT image. $S$-values can also be calculated for patient-specific geometries using voxel-based Monte Carlo methods and a CT image to derive the tissue properties [91]. For 177Lu, the $S$-value from a source voxel $h$ to a target voxel $k$ can be expressed:

5 $${S}_{177\mathrm{Lu}}\left(k\leftarrow h\right)= \frac{1}{{m}_{k}} \left[\sum_{i \in 177\mathrm{Lu},\mathrm{e}}{E}_{i} {Y}_{i} {\phi }_{\mathrm{e}}\left(k\leftarrow h,{E}_{i}\right) +\sum_{i \in 177\mathrm{Lu},\mathrm{ph}}{E}_{i} {Y}_{i} {\phi }_{\mathrm{ph}}\left(k\leftarrow h,{E}_{i}\right) \right] ,$$

where ${m}_{k}$ is the target voxel mass, and the AF has been separated into components for electrons and photons. Using the LED approximation for self-absorbed dose gives ${S}_{177\mathrm{Lu}}\left(k \leftarrow h\right)\approx {\Delta }_{177\mathrm{Lu},\mathrm{e}}/{m}_{k}$; ($k=h$). As for region-based dosimetry described above, application of the LED approach for voxel-based 177Lu dosimetry is motivated by the short electron ranges in soft tissue and bone, compared to the voxel dimensions of the SPECT images. In addition, due to the limited spatial resolution of contemporary SPECT systems, blurred estimates of the real underlying activity distribution are produced, thus limiting the spatial scale that can be accurately resolved. The error in assuming LED is in many cases considerably smaller than that introduced by the spatial blurring of the activity distribution [92]. From Eq. 5 and assuming LED, the self-absorbed dose rate to voxel $k$ can be calculated based on the voxel activity volume-concentration ${\left[A\right]}_{k}$ derived from a quantitative SPECT image, according to

6 $${\dot{D}}_{k,\mathrm{self}}\left(t\right)= {\Delta }_{177\mathrm{Lu},\mathrm{e}} \frac{{\left[A\right]}_{k}\left(t\right)}{ {\rho }_{k}} .$$

When a voxel-wise map of the mass density ${\rho }_{k}$ is not available, assuming a uniform mass density for soft tissue is often sufficient. For bony structures and tumours located in these tissues, other density values are required.

Equation 6 is applicable when the contribution from cross-absorbed dose is low and for mid-size source/target regions in soft tissue or bone, with comparatively convex shapes. Curve fitting of the absorbed dose rate distribution versus time can be applied at the voxel level to obtain an absorbed dose map. Alternatively, fitting can be applied to the mean or median absorbed dose rates in a volume of interest (VOI). The former option allows for visual inspection of the absorbed dose distribution and the construction of dose-volume histograms (DVHs). However, DVHs are recognised to be sensitive to noise, limited spatial resolution, and requires that co-registration is applied to the time series of SPECT images which can introduce undesired interpolation effects. VOI-based voxel dosimetry basically represents an alternative route of dosimetry as per Eq. 3.

### Factors that modify the radiobiological response

Different activity uptakes and excretion rates can produce the same absorbed dose, although the absorbed dose rates differ. In EBRT and brachytherapy, the absorbed dose rate is known as a modifying factor for the radiobiological effects, owing to cellular repair during radiation exposure. [177Lu]Lu-SSRT or [177Lu]Lu-PSMA therapies are characterised by low absorbed dose rates in comparison to most other radiotherapy techniques. Fractionation is another factor associated with cellular repair and tissue recovery, especially for late-responding tissues. This is considered in therapies with [177Lu]Lu-SSRT or [177Lu]Lu-PSMA, which are generally given in repeated cycles (or fractions), with a pre-defined cycle interval. An additional modifying factor is nonuniform radiation exposure, which, owing to the short electron range of 177Lu, is characteristic for these therapies.

The biologically effective dose ($\mathrm{BED}$), more recently included as a special case of the equieffective dose, was introduced in the linear-quadratic (LQ) model to quantify the different absorbed doses required to induce a given radiobiological effect [93, 94]. The $\mathrm{BED}$ takes into account the total absorbed dose $D$, the absorbed dose rate, effects of repair and fractionation. It is specific for the tissue and the considered radiobiologic endpoint for which the LQ-model parameter $\alpha /\beta$ was derived. The $\mathrm{BED}$ is formulated as a double integral that specifies the interaction of the rate of tissue-damage induction due to radiation exposure and the rate of repair. For a single radionuclide-therapy administration and assuming a mono-exponential washout, this double integral evaluates to [95]

7 $$\mathrm{BED}= D\cdot \left(1+\frac{D}{\alpha /\beta }\cdot \frac{\lambda }{\lambda +\mu }\right)$$

where $\lambda$ is the rate constant linked to the effective half-life of the radiopharmaceutical in the tissue $\left(\lambda =\mathrm{ln}2/{T}_{\mathrm{eff}}\right)$, and $\mu$ is the repair constant, assuming a mono-exponentially decreasing rate of repair. The BED expression has been extended to the MIRD schema and applied to organs at risk such as the red marrow and kidneys [58, 59]. BED expressions were derived for fractionated treatments, which for fractions separated by long time intervals with respect to the effective half-life result in the sum of the BED from each fraction [96, 97].

## Quantification of 177Lu activity

### Calibration of the activity metre

Prior to delivery of any treatment, the activity metre, also called dose calibrator, should be correctly calibrated for the containers used for dispensing the activity. It needs to be assured that the measured activity is traceable to a primary standard. This can be achieved by calibration of the activity metre dial settings towards a 177Lu source that is accompanied by an activity statement with traceability to a standard metrology laboratory [98]. The stability of the activity metre response also needs to be monitored.

### Quantitative SPECT/CT imaging

177Lu is one of the best-characterised radionuclides with regards to image-based activity quantification. Whilst early dosimetry studies were predominantly based on planar gamma-camera images, SPECT imaging is for many applications now considered the method of choice [99, 100]. Quantitative SPECT/CT is described in MIRD pamphlet 23 [101] and the EANM/MIRD guideline for quantitative 177Lu SPECT [102]. The intention in the following is to summarise the practical steps most relevant to dosimetry for 177Lu-labelled compounds.

#### Camera calibration factor

Camera calibration refers to the process used to convert the counts measured by the SPECT camera to activity. The calibration factor is determined by imaging a source of known activity, or activity concentration, in a reference geometry, using the same SPECT system and acquisition settings as used for patient imaging. Currently, the source geometry and imaging parameters are not standardised, and different approaches have been reported for 177Lu [100, 103–108]. Of the methods proposed, a large phantom, similar to that used for PET image calibration, is considered the most robust calibration geometry. The SPECT calibration factor ${Q}_{\mathrm{sp}}$ is derived from the tomographic image, reconstructed using the same protocol as used for patient imaging. ${Q}_{\mathrm{sp}}$ is defined as the reconstructed count rate per activity (cps/MBq), according to

8 $${Q}_{\mathrm{sp}}=\frac{{C}_{\mathrm{cal}}}{{A}_{\mathrm{cal}}} ,$$

where ${C}_{\mathrm{cal}}$ is the count rate in a VOI, calculated as the total counts in the VOI divided by the acquisition time-interval. The denominator ${A}_{\mathrm{cal}}$ is the product of the activity concentration in the phantom and the volume of the VOI. Some commercial systems have introduced quantitative reconstruction algorithms that produce images in the unit of activity, or activity concentration, instead of counts. Calibration of such systems is still based on physical measurements, and the calibration factor requires verification. Calibration factors may also vary over time, depending on the stability of the system or if the camera is re-tuned, and repeated monitoring is advised.

#### Count rate performance

Effects of pulse pile-up and dead time should be considered when imaging during therapies with [177Lu]Lu-SSRT or [177Lu]Lu-PSMA if the count rate is expected to be high at patient imaging. Such effects render the camera system to respond nonlinearly to the activity in the field-of-view (FOV) [102, 109]. Characterisation of the count-rate performance is made by imaging of a range of activities, covering the maximum activity likely to be encountered in the patient. The geometry needs to be chosen such that the amount of scatter is similar to that of patient imaging, for example by using a source within a large cylindrical phantom. The magnitude of the dead-time effect depends on the total count rate incident across the entire energy range. Therefore, it is also dependent on the scattered events incident on the detector. As scatter and count rate can vary at different projection angles, dead time effects will also vary with the projection angle [101]. A dead-time correction factor has been developed for 131I SPECT, which is based on the mean count rate overall projections [110]. Following the current standard administration protocol of 7.4 GBq of 177Lu per cycle and recognising that there is an initial component of fast urinary excretion of [177Lu]Lu-SSRT and [177Lu]Lu-PSMA, dead time effects are generally only of concern within the first hours after administration and less for kidney and tumour dosimetry [109]. For higher activity administrations, correction for count-rate performance may be necessary.

#### Correction for the partial-volume effect (PVE)

The PVE is relevant in most cases of 177Lu dosimetry. It is essentially a result of the limited spatial resolution of SPECT systems, which produces a blurred version of the underlying activity distribution. Assessment of PVEs can be made by phantom studies of a set of inserts covering a range of clinically relevant volumes. The same parameters for image acquisition and reconstruction as used in patient studies are applied. The recovery coefficient $R(v)$ of an insert of volume $v$ is calculated according to

9 $$R\left(v\right)=\frac{{C}_{\mathrm{R}}\left(v\right)}{{Q}_{s\mathrm{p}}\cdot {A}_{\mathrm{R}}\left(v\right)} ,$$

where ${C}_{\mathrm{R}}(v)$ is the count rate measured in a VOI of volume $v$, and ${A}_{\mathrm{R}}(v)$ is the 177Lu activity contained in the insert at the time of measurement. Alternatively, Eq. 9 can be formulated in terms of activity concentration and count rate concentration. Commonly, spherical inserts are used to determine recovery coefficients [72]. It may also be appropriate to characterise the recovery curve using nonspherical objects, or with different source-to-background contrast ratios. For kidney-shaped objects, recovery coefficients have been measured using 3D-printed objects or Monte Carlo simulated images [106, 111, 112]. For PET/CT, a number of image-based correction methods have been developed that may also be applicable to 177Lu dosimetry [113].

#### Patient image acquisition and image processing

It is recommended to use the same SPECT/CT system for the entirety of the dosimetry study. Medium energy collimators are recommended for 177Lu imaging for most systems. Although 177Lu has two photo peaks (113 and 208 keV), commonly only the 208 keV photopeak is used for quantitative imaging with NaI-based gamma cameras, as this peak contains considerably less scatter than the 113 keV window [102]. For systems based on CZT crystals, the 208 keV peak may be outside the spectral range, and the use of the 113 keV peak has been investigated [114]. On NaI-based cameras, an energy window of 15 to 20% is common for the 208 keV peak, and when the triple-energy window (TEW) scatter correction is employed, two additional, narrow scatter windows are set adjacent to the main window. The camera should be set to automatic contouring and projections acquired in a 128 × 128 matrix or higher (zoom factor = 1). Iterative tomographic reconstruction is strongly recommended, including CT-based corrections for attenuation, scatter, and, when available, collimator-response modelling (also termed resolution recovery). The number of projections and time per projection should be chosen based on the expected signal-to-noise ratio of the VOI counts, which is governed by the amount of activity in the patient, the camera system sensitivity, the matrix size, and the noise propagation of the tomographic reconstruction. Between 60 and 120 projection angles are generally recommended, although, for estimation of the activity concentration in centrally located, high-uptake tissues, the number of projections can potentially be reduced [115]. Scan times vary widely but are typically in the order of 30–40 s per projection [116] and can be adjusted between early and late imaging time points. For [177Lu]Lu-PSMA therapies, multiple-FOV SPECT/CT may be required to cover the entire extent of the disease. For this reason, shorter times per projection have been investigated [115], possibly opening for near whole-body SPECT imaging. The number of updates (iterations $\times$ subsets) should be higher for quantitative imaging than for diagnostics, as the main purpose is to obtain a reliable estimate of the activity in source regions. The reconstruction protocol for activity quantification should be optimized to ensure convergence of the VOI count rate [102, 106]. As a first approach, the phantom used for recovery measurements can be used to examine the rate of convergence. The application of post-reconstruction filtering is not recommended for quantitative imaging, as this will affect the recovery and thus the quantitative accuracy.

#### Image analysis for activity quantification and absorbed dose calculation

Quantification of the activity $A\left({r}_{S},t\right)$ in a source region at time $t$ post administration is made based on the total count rate $C({v}_{\mathrm{VOI}},t)$ measured within a VOI of volume ${v}_{\mathrm{VOI}}$ over the source region, according to

10 $$A\left({r}_{S},t\right)=\frac{C({v}_{\mathrm{VOI}},t)}{{Q}_{s\mathrm{p}}\cdot R\left({v}_{\mathrm{VOI}}\right)} .$$

A robust and consistent segmentation strategy needs to be maintained for VOI delineation. Furthermore, the segmentation method needs to be applicable for all imaging time points and across patients. The practical implementation of SPECT image segmentation depends partly on the image data available. For hybrid SPECT/CT systems, VOI delineation for organs is preferably made using CT information. Due to organ motion between the CT and SPECT acquisitions, the VOI positions may require adjustment to the SPECT data set. For tumours, VOI delineation on a low-contrast CT is often challenging, and a co-registered contrast-enhanced CT or the SPECT image may then be useful. Techniques used for SPECT image segmentation include manual operator delineation, fixed-percentage thresholds [72], adaptive or automated thresholding [117, 118], gradient-based surface adaption [119], and methods based on convolutional neural networks [120]. Fixed thresholding on SPECT images is commonly available in commercial systems but has the disadvantage of being highly sensitive to local contrast and noise [121]. The estimation of the mass of the target region $m\left({r}_{T}\right)$ can be made from segmentation in PET, CT, or MRI images, or, depending on the segmentation strategy, based on the same VOI as applied to the SPECT images.

The absorbed dose can be reported for whole organs, tumours, or parts of these, although the limitations associated with spatial resolution and noise need to be respected. Segmentation of parts of large organs, such as the liver, may be useful to assess regional differences in absorbed dose if there are pronounced clusters with different activity concentrations.

### Planar image-based activity quantification

Although planar imaging is known to suffer from superposition of activity in tissues that lie above or below the source region to be analysed, there are still applications within 177Lu dosimetry. These include estimation of the total-body TAC for tissues where the cross-absorbed dose from the photons emitted by 177Lu is important, as may be the case for bone-marrow. Other applications include dosimetry for salivary, lacrimal and pituitary glands. Planar image-based activity quantification has been described in MIRD Pamphlet 16 [122], and the intention is to summarise the methodological aspects most relevant to dosimetry for 177Lu-labelled compounds.

#### Camera calibration factor

Earlier methods for planar image-based activity quantification were based on patient acquisition early after administration before the patient had voided. A conversion factor was calculated from the image counts over the whole body divided by the administered activity. This conversion factor was then assumed to take all physical effects into account, i.e. both the system sensitivity and effects of photon attenuation and scattering, thus neglecting the variation in these phenomena across the patient body.

A preferable method is to determine the calibration factor separately and then apply corrections for attenuation and scatter. The planar calibration factor, ${Q}_{\mathrm{pl}}$, represents the count rate obtained per unit of activity for a source placed in the air, determined by planar image acquisition of 177Lu with a known amount of activity [123, 124]. To determine ${Q}_{\mathrm{pl}}$, a region-of-interest (ROI) is delineated around the source, with a margin to take the resolution-induced spill-out into account, and the sum of the ROI counts is divided by the acquisition time interval and the source activity. The time interval should represent the time that a particular pixel is in the camera FOV at patient imaging, and when whole-body scanning is used, the calibration measurement may need to be made in scan mode, depending on how the acquisition time is reported in the DICOM header. For dual-head cameras, calibration image acquisition needs to be made for both camera heads, and when applicable, the geometric mean of the counts taken. A long background scan can also be made to assess the impact of imperfect nonuniformity correction and examine the background count rate. Preferably, scatter correction should be applied before the determination of the ROI count rate.

#### Patient image acquisition, image processing, and analysis

Regarding the collimator and energy window settings, the same recommendations apply for planar as for SPECT image acquisition. The matrix size is often 1024 × 256, covering the patient’s length. The couch velocity is adjusted to the expected count rate at patient imaging and may vary for the different time points after administration.

The conjugate-view method is the most commonly used method for activity quantification from anterior-posterior planar images [122, 125, 126]. The activity in a source region is calculated according to

11 $$A\left({r}_{S},t\right)=\sqrt{{C}_{\mathrm{A}}\left(t\right){\cdot C}_{\mathrm{P}}\left(t\right) }\cdot a\left(\mu ,L\right)\cdot \frac{1}{{Q}_{\mathrm{pl}}} ,$$

where ${C}_{\mathrm{A}}(t)$ and ${C}_{\mathrm{P}}(t)$ are the count rates in ROIs delineated over the source region in the anterior and posterior images, respectively. The attenuation correction $a\left(\mu ,L\right)$ is given by $\mathrm{exp}\left(\mu \cdot L/2\right)$, where $L$ is the patient thickness at the source-region location, and $\mu$ is the attenuation coefficient for the 208-keV emission (assuming that the energy window is set over this photopeak). An additional factor is sometimes used in Eq. 11 to correct self-attenuation in the source region [122, 125, 126]. However, this factor becomes near unity for 177Lu, and it can thus be omitted. The simplest method for attenuation and scatter correction is to use an effective, or broad-beam attenuation coefficient, ${\mu }_{\mathrm{eff}}$. The value of ${\mu }_{\mathrm{eff}}$ needs to be determined experimentally and has for 177Lu (208 keV) been reported to approximately 0.12 cm^-1^ [127]. The attenuation-correction factor $a\left(\mu ,L\right)$ can also be derived from a transmission scan of the patient, by use of a 57Co flood source or a CT localizer [128, 129]. As the obtained attenuation map is valid for the transmission energy, either 122 keV (57Co) or the mean energy of the X-ray spectrum (CT localizer), it needs to be scaled to the emission energy for 177Lu [129]. Such transmission-based methods enable the calculation of an attenuation map $a\left({\mu }_{x,y},{L}_{x,y}\right)$, which takes the spatially varying patient thickness and attenuation over the body into account. Scatter correction can be implemented using the TEW method [130] or using model-based methods [131].

ROIs are delineated in the images from each time point. Alternatively, the series of patient images can be co-registered prior to ROI delineation as this enables propagation of ROIs between images from different time points. When there are pronounced activity uptakes in under- or over-lying tissues, background correction needs to be applied [132]. For [177Lu]Lu-SSRT and [177Lu]Lu-PSMA, the plasma turnover is generally fast, rendering the plasma background comparably low for later acquisition time points, although overlap from physiological uptake and tumours still need to be considered. Before the application of Eq. 11, the count rates in background ROIs are then subtracted from ${C}_{\mathrm{A}}(t)$ and ${C}_{\mathrm{P}}(t)$, delineated at a location that represents the concentration of the overlapping tissue and with a similar body thickness as over the source region [122, 132].

### Hybrid planar–SPECT/CT activity quantification

The combination of planar and SPECT/CT-based activity quantification has been implemented as an alternative to repeated SPECT/CT imaging [133]. TACs in relative units are determined for the relevant source regions from the planar scans acquired at several time points. The amplitude of the TACs is then rescaled using the source-region activity quantified in an absolute unit from a SPECT/CT acquired at the one-time point. As the purpose of the planar images is to capture the shape of the TAC rather than its amplitude, effects of attenuation and scatter are expected to be modest. The ROIs can be smaller than those used for whole-organ activity estimation, so as to avoid regions with extensive overlap that may otherwise generate errors in the estimated TAC shape. ROIs should be placed in the same part of the source region in the images from different time points, and any differences in ROI size, or scan speed, taken into account. Background correction can be applied if there is relevant interference from overlapping tissues that may affect the TAC shape.

## Estimation of the time-integrated activity

The TIA is calculated from the area under the TAC, either through trapezoid integration or by analytic integration of a function fitted to the data. For either approach, the activity needs to be measured periodically, post administration of the radiopharmaceutical.

### Time sampling

The timing of activity measurements must be carefully chosen to adequately characterise the pharmaceutical uptake, retention, and washout. In most cases, the TAC can be described by a sum of exponential functions. Ideally, a minimum of three data points should be used to define each exponential phase. There is substantial work within the literature exploring optimum timing regimens for therapies with [177Lu]Lu-PSMA and [177Lu]Lu-SSRT, particularly focusing on the pharmacokinetics of the kidney. Characterisation of the early-phase kinetics of [177Lu]Lu-DOTA-TATE was achieved by dynamic scintigraphy for the first 60 min post-administration [134]. It was noted that although the early fast phase did not contribute substantially to the estimated renal absorbed dose, it could detrimentally influence the evaluation of the effective half-life. It is therefore generally accepted to make image acquisition after 4 h and assume instantaneous uptake rather than perform early imaging and risk misrepresenting the organ TAC. Late time point imaging was also investigated by imaging up to 10 weeks post-administration [4]. The most important finding was kidney and spleen uptake in images acquired up to a month after treatment and tumour uptake visualised up to 7 weeks after injection. The total body TAC had a notable tail, which was not completely captured by imaging during the first week. The absorbed doses to total body and tumours obtained when including these late time points were on average 5–6% higher than those obtained when only using data acquired during the first week. A recent review on dosimetry for therapies with [177Lu]Lu-SSRT or [90Y]Y-SSRT showed that in all cases a minimum of 3-time points were acquired (3 in 44%, 4 in 56%), the most common were on the day of administration (between 1 and 4 h), the following day, at day 2 and day 7. Imaging on days 3 and 4 was also common [135].

### Curve fitting

**Fig. 2:**
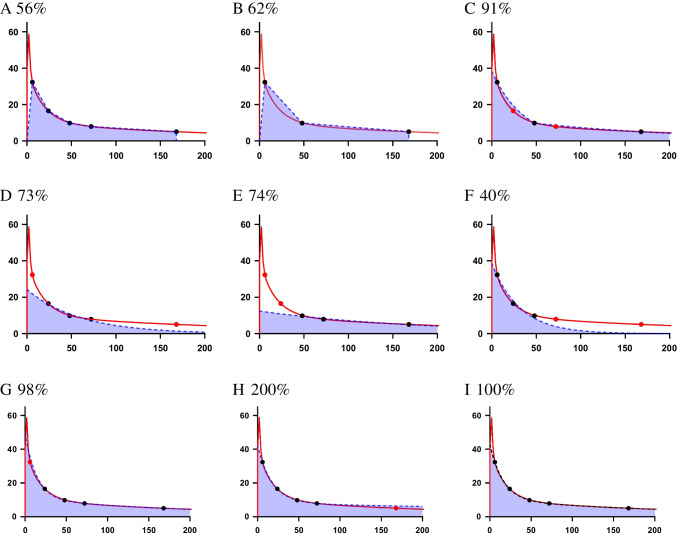
Quantification of the TIA from a hypothetical TAC. The red line describes the true, underlying activity retention. Black dots indicate activity measurements performed at nominal times of 5, 24, 48, 72, and 168 h. Red dots indicate measurements that were omitted from this nominal scheme. Panels A–I illustrate different sampling and fitting scenarios and also include the estimated TIA as a percentage of the reference TIA. **A**–**C** trapezoidal integration based on 5 (**A**), or 3 data points (**B**, **C**) where **C** includes extrapolation beyond last data point; **D**–**F** integration based on mono-exponential curve fitting to 3 data points at different times; **G**–**I** integration based on bi-exponential curve fitting to 4–5 data points

The accuracy of the TIA estimate will depend on the characterisation of the TAC. Figure 2 illustrates the general effects of time sampling and TIA calculation when applied on an arbitrary, hypothetical TAC. The true, underlying activity retention has been formed as a sum of exponential functions with both uptake and multiple decay phases. The integral of this function from $t=0$ to infinity is taken as the reference TIA.

For trapezoidal integration, in Figure 2(A–C), the data are interpolated linearly between points, and the more data available, the better the interpolation fits the true curve. In A and B, the TIA contribution beyond the last data point is not estimated. Further improvement can be made by assuming physical decay or extrapolating the data to infinity using the last two measurements (C). This approach is marginally more complex but arguably results in a better estimate of TIA than the simple linear interpolation. When sufficient data points exist, it is possible to use ordinary least squares to fit an appropriate function to the dataset (D–I). In examples (D–F), a monoexponential function is insufficient to adequately represent the true data, and the errors in the TIA vary greatly depending on which time points are used. This is especially true in Figure 2F, where the late phase of the TAC is not characterised. It is evident from Figures G, H, and I that fitting a bi-exponential function gives a good representation of the true TAC. In each case, the early spike due to fast wash-in and wash-out is not characterised. However, the area under the spike contributes little to the total TIA and is considered negligible. The importance of properly measuring a later time point is demonstrated in Figure H, where the slow late phase is slightly overestimated but results in an overestimate of the TIA by a factor of 2. The method of integration and the assumptions made regarding uptake or excretion before the first time point or following the last time point can have a significant impact on the TIA calculations and should thus be addressed carefully.

Random and systematic errors will also affect the accuracy of the TIA estimate. Trapezoid methods will be more influenced by these errors. The fitting of analytical functions to the data will be less influenced by random errors, which can be further reduced by increasing the number of data points acquired. Advanced techniques for deriving the appropriate TAC function include statistical tests [136] and pharmacokinetic modelling [137] that can potentially provide a biological basis of fitting functions. Methods for determining the uncertainty in the fit parameters and the TIA are described in EANM guidance [138].

### Single time point

**Fig. 3:**
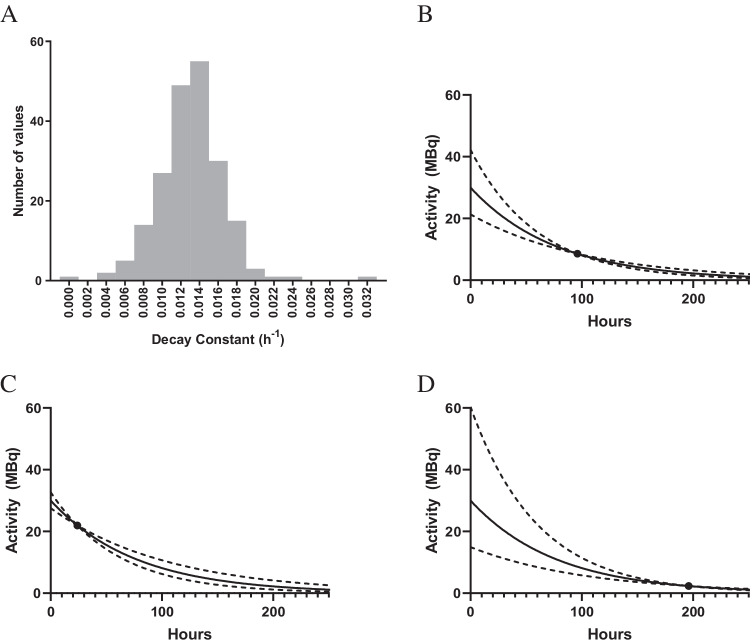
**A** Histogram of effective decay constants for a patient population with a mean of 0.013 h−1 and coefficient of variation of 27%. **B**–**D** Solid lines show TACs estimated from an activity measurement at 96 h (**B**), 24 h (**C**), and 196 h (**D**) combined with the mean population half-life. Dotted lines indicate the standard uncertainty in the respective TAC, yielding relative uncertainties in the TIA that are up to 10% for 96 h (**B**), 26% for 24 h (**C**), and 60% for 192 h (**D**)

It is generally not recommended to routinely perform absorbed dose calculations based on a single time point. However, as shown in recent publications on [177Lu]Lu-SSRT and [177Lu]Lu-PSMA therapies [139–142], it is possible if the pharmacokinetics has previously been characterised across a population, and the effective half-life in the organ of interest does not vary widely from patient to patient. The uncertainty associated with such techniques is governed by the distribution and representativeness of the population data. The influence of timing and use of population-based half-lives in single-time point dosimetry is illustrated in Figure 3. The distribution of the decay constants is used to determine the population mean and coefficient of variation. These are used to determine the relative uncertainty in the estimated TIA when combined with measurements made at different times. Errors and uncertainties associated with the activity measurement will add to the uncertainty of the TIA, and the assumption of a single exponential will not always be appropriate.

## Dosimetry of organs, tissues, and tumours

### Extravasation and absorbed doses to the skin

#### Summary of available dosimetry data

When high activities are administered through a punctured vein, potential extravasation of 177Lu may lead to high absorbed doses and injury of surrounding tissue, such as skin desquamation and necrosis [143]. The absorbed dose to the site of injection would be very high if clearance had not occurred. Consider a volume of 100 cm^3^ filled with 7.4 GBq: without clearance, this would lead to an absorbed dose in the order of 1400 Gy. However, neither for [177Lu]Lu-SSRT [144–146] nor [177Lu]Lu-PSMA therapies [147, 148] any serious adverse reactions have been observed from extravasation and none of the reported cases required aggressive interventions. Rapid clearance from the extravascular space by lymphatic drainage, with a typical clearance half-life from the arm of 1.2–3 h, has been observed for both compounds [144–146]. Absorbed doses to the surrounding tissue of 6–10 Gy were reported, all below the 20 Gy threshold for ulceration and permanent skin breakdown [149, 150]. The absorbed dose to the basal epidermal layer was estimated to be 3 Gy [144].

#### Methodological aspects and recommendations

Extravasation monitoring is often made by whole-body planar imaging. However, the risk of false negatives should be considered in cases where the patient’s arm is on the border of the camera FOV. A gamma-probe can be used to measure the count rate at the injection site, enabling relative measurements and an accurate determination of the rate of washout. In case of extravasation, SPECT imaging is advisable to determine the volume of the infiltrated tissue and to quantify the activity. Typically, two or three imaging time points at 2, 4, and 24 h are sufficient to estimate the TAC. Alternatively, one SPECT image at an early time-point can be used in conjunction with probe measurements at the injection site. The estimated absorbed doses will in most cases be uncertain due to difficulties of determining the exact volume of the infiltrated tissue. A relevant range of volumes can be applied to assess the uncertainty in the estimated absorbed dose.

### Dosimetry for the kidneys

The kidney is probably the most well-examined organ in terms of dosimetry for 177Lu therapy. For both [177Lu]Lu-SSRT and [177Lu]Lu-PSMA therapies, there is renal transit, and especially for [177Lu]Lu-SSRT also renal accumulation, making the kidneys a treatment-limiting organ. Dosimetry-guided treatments have been applied that tailor the number of therapy cycles and/or the administered activity per cycle with respect to the renal absorbed dose. The intent is then to achieve the maximum possible absorbed dose to tumour while respecting constraints for the kidneys.

#### Summary of available dosimetry data

The kidney absorbed dose levels in therapies with [177Lu]Lu-SSRT and [177Lu]Lu-PSMA are of a similar magnitude. Mean absorbed doses range between 0.54 and 1.00 Gy/GBq for [177Lu]Lu-SSRT [36, 151] and between 0.4 and 0.8 Gy/GBq for [177Lu]Lu-PSMA [34].

The ranges of the reported values can be associated with different patient characteristics such as renal function and tumour burden, the use of different amino-acid solutions for renal protection, and methodological aspects, such as the selection of imaging time points and methods used for activity quantification and dosimetry. The variability due to patient-specific factors is reflected by the reported absorbed dose ranges from single centres. For [177Lu]Lu-PSMA, the minimum and maximum absorbed doses vary with up to a factor three [12, 21, 23] and even up to a factor of 9 (range 0.09–0.84 Gy/GBq) [74]. For [177Lu]Lu-SSRT, similar inter-patient ranges have been reported from single centres, for example 0.3–1.98 Gy/GBq [36, 55]. Clinical trials that employ kidney dosimetry to tailor the number of [177Lu]Lu-SSRT cycles to the individual patient show a wide variation (2–10) between patients [55, 152]. Similarly, the total administered activity required to reach a pre-specified absorbed dose limit has been reported to vary across patients, by a factor of 1.5 ([177Lu]Lu-SSRT) or even a factor of 3 ([177Lu]Lu-PSMA) [21, 153].

There is also an intra-patient variability in the absorbed doses across therapy cycles with [177Lu]Lu-SSRT [55, 99, 127, 154–157] and [177Lu]Lu-PSMA [12, 23]. In the majority of patients (80%), the absorbed dose per unit of administered activity is within 30% of the previous cycle. However, differences of up to a factor between 2 and 3 have been reported, possibly due to tumour response during the cycles or changes in the renal function [55, 155].

Owing to the inter- and intra-patient variability in the absorbed dose per unit of administered activity, administering a fixed amount of activity across a fixed number of therapy cycles results in a highly variable cumulative kidney absorbed dose.

#### Methodological aspects

Kidney dosimetry based on planar imaging has been shown to overestimate or result in less precise absorbed dose estimates compared to dosimetry based on quantitative SPECT/CT [99, 154, 158, 159]. Studies that compared planar- and SPECT-based dosimetry in the same cohort of patients arrived at different conclusions, possibly depending on the methods used. Deviations of between 10 and 300% were observed, or different deviations for the right and left kidney [99, 158, 159]. Presently, kidney dosimetry based on SPECT/CT is regarded as the preferred method. Hybrid planar-SPECT/CT methods have also been demonstrated as a viable option [99, 154, 158, 159].

For patient-specific dosimetry, estimation of the kidney mass is a pre-requisite, commonly based on CT volumetry and assuming a mass density of 1.05 g/cm^3^ [160]. The mass has been shown to vary between patients, up to a factor three [12, 23, 127, 151, 159, 161]. The contribution to the kidney absorbed dose from the rest of the body has been shown to be low (approximately 2% of the absorbed dose), and consideration of only the self-absorbed dose is sufficient [127].

SPECT image segmentation and VOIs should preferably encompass the kidney regions that retain the activity over an extended period, i.e. the cortex and medulla. Ideally, the VOIs should be delineated based on the CT images acquired as part of the SPECT/CT study. However, a simplified approach has been proposed where a small VOI is placed in the centre of the organ [152, 159]. When compared with whole-organ segmentation in low-resolution SPECT images, i.e. including a post-reconstruction filter and attenuation correction, average underestimations of the mean kidney absorbed dose of 8% (left) and 14% (right) were obtained, with differences ranging from −55% to +78% [159]. Subsequent studies adopted this method as it has the advantage of fast execution [151, 153, 157, 162]. However, when applied to SPECT images with improved spatial resolution, i.e., including corrections for attenuation, scatter, and resolution-recovery, the renal absorbed doses were considerably higher than those derived by whole-kidney segmentation, with a mean factor of 1.8 [163]. Automatic segmentation based on machine learning has been applied for kidney dosimetry of [177Lu]Lu-PSMA and [177Lu]Lu-SSRT and demonstrates a minor deviation (3%) in absorbed dose estimates compared to manual segmentation [120].

Partial-volume correction is required for kidney SPECT/CT quantification since the PVEs can be considerable. The recovery coefficient should be estimated with respect to the adopted methods for image reconstruction and segmentation, in phantom studies of kidney-shaped objects. Typical recovery coefficients for kidneys have been reported to lie between 0.80 and 0.90 [111, 112]. With a mean renal-cortex thickness of 6–7 mm [164], the spatial resolution of current SPECT systems limits the possibility to separate activity in the respective renal sub-regions cortex and medulla. Possibly, future SPECT/CT systems will offer improved spatial resolution and allow for clinical kidney dosimetry on a finer scale [165, 166].

The number of acquisition time points used to estimate the renal TIA affects the accuracy of the estimated absorbed doses [122, 127, 167]. In the majority of patients, the biodistribution of [177Lu]Lu-SSRT and [177Lu]Lu-PSMA is characterised by a fast initial plasma washout, commencing at infusion and lasting a few hours after administration, followed by exponential washout. Most studies report the use of at least one image acquisition during the first day (typically 4 h) after administration, one acquisition at 24 h, and then between one and three acquisitions at later times. A monoexponential function is generally used to characterize the wash-out phase [12, 135, 151]. The impact of acquisitions up to 7 days after administration has been emphasised [127, 167, 168]. Furthermore, it has been shown that the early acquisition (before 24 h) has a minor impact on the absorbed dose estimate, and due to the fast plasma washout phase, a very early time point (<4 h) should be avoided for estimation of the TIA [134, 162].

For the mono-exponential washout phase, a mean (or median) effective half-life of approximately 50–60 h has been reported, although considerable inter-patient ranges have been observed [55, 151, 169]. For the individual patient, the effective half-life is generally consistent between cycles, thus supporting the application of the effective half-life measured in one cycle across subsequent cycles. This method is justifiable as long as there are no clinical reasons to expect changes in the renal accumulation, such as changes in kidney function, the accumulation of the amino acid used for renal protection, or relevant tumour response or growth. Notably, considerable intra-patient variability has also been reported, with variations by up to a factor three [55, 151, 169]. Verification and re-assessment of the half-life may therefore be warranted.

#### Recommendations for kidney dosimetry

The recommended protocol for kidney dosimetry is as follows: Image acquisitions are made using SPECT/CT centred over the kidneys. At the first therapy cycle, three acquisitions are made between day 1 (24 h after administration) and day 7, with at least 2 days between the last two time points and preferably between all time points. A less preferable but sometimes more feasible alternative is SPECT/CT acquisition at a one-time point complemented by planar imaging. For subsequent cycles, a minimum of one SPECT/CT is made, preferably at a time that corresponds to the intermediate time point of the first cycle. It is advisable to maintain a consistent acquisition time for the patient across the cycles, so as to enable comparison of data. SPECT/CT image segmentation is preferably based on the CT, with a VOI that encompasses the entire cortex and medulla.

Absorbed dose calculation is implemented through a sequence of steps, which can be taken in a different order and be applied on the level of regions or voxels (Eqs. 1–6). Table 3 demonstrates two example schemes for absorbed dose calculation.

**Table 3:** Calculation of the mean absorbed dose to the kidneys, following two example schemes

Example 1	Example 2
The activity (MBq) in each kidney is determined from the respective VOI drawn on the SPECT image and application of Eq. 10.	The mean/median activity concentration (MBq/mL) in each kidney is determined from the respective VOI drawn on the quantitative SPECT image. The recovery coefficient is applied to the mean/median activity concentration.
Cycle 1: A mono-exponential curve is fitted to the time-activity data to determine the patient-specific effective half-lives for the left and right kidney.	The mean/median absorbed dose rates for left and right kidneys (mGy/h) are calculated by multiplication of the activity concentration with ${\Delta }_{177\mathrm{Lu},\mathrm{e}}$ (Table 1) and division by a mass density of 1.05 g/cm3 (Eq. 6).
Calculation of the TIA (MBq h) for the left and right kidney by analytical integration over time: Cycle 1: based on the parameters of the fitted curve. Remaining cycles: based on a combination of the cycle-specific activity and the patient-specific half-lives for left and right kidneys. The TIA representing both kidneys is calculated as the sum of the TIA for the respective kidney.	Cycle 1: A mono-exponential function is fitted to the time-dose rate data to determine the patient-specific effective half-lives for the left and right kidney.
The mass of each kidney is calculated from the VOI volume multiplied by a mass density of 1.05 g/cm3. The total kidney mass is calculated as the sum of the left and right kidney mass. The $S$-value is scaled to the patient-specific value (Eq. 4).	Calculation of the absorbed dose (mGy or Gy) for the respective kidney by analytical integration over time: Cycle 1: Based on the parameters of the fitted curve. Remaining cycles: based on a combination of the cycle-specific absorbed dose rates and the patient-specific half-lives for the respective kidney.
The mean absorbed dose for the left and right kidney (mGy or Gy) is calculated (Eq. 3) using the patient-specific (mass-adjusted) $S$-value and the sum of the TIA for both kidneys.	The mean absorbed dose for the left and right kidney is calculated as the (mass-weighted) mean value.

### Dosimetry for bone marrow and blood elements

The radiosensitivity of the bone marrow is associated with the red-marrow cells, which are thus considered the target region in bone-marrow dosimetry. These cells are located in skeletal cavities among other structures such as trabecular bone and inactive marrow (adipose tissue). $S$-values for red marrow thus rely on models of its distribution within the cavities [170]. In 177Lu therapy, radiation exposure of the red marrow is caused by activity in the marrow itself, bone, other organs with high uptake, and the total body.

#### Summary of available dosimetry data

Dosimetry for the red marrow is not routinely performed in 177Lu therapies. However, several studies reported red marrow absorbed doses for [177Lu]Lu-SSRT with a median value across all studies of 50 mGy/GBq (range: 2–150 mGy/GBq) [6, 31, 42, 99, 152, 171–173]. The largest study included 176 patients and reported a median value of 16 mGy/GBq, interquartile range 12–22 mGy/GBq [152]. The median absorbed dose for [177Lu]Lu-PSMA is comparable at 44 mGy/GBq (range: 10–340 mGy/GBq) [12, 21, 23, 74, 174–176]. In most prostate cancer patients, [177Lu]Lu-PSMA uptake in skeletal metastases influences the bone-marrow absorbed dose, and considerably higher values with a median (range) of 100 (10–340) mGy/GBq have been reported for such cases [74].

The total-body TAC for [177Lu]Lu-SSRT is generally biphasic, with effective half-lives of 1.3 (0.9–1.5 h) and 50 h (45–57 h) [152], although this may depend on the tumour burden. Also, the blood TAC follows a biphasic pattern, where the first phase has an amplitude of 2.6 ± 1.4 %IA/L (94% of total) with an effective half-life of 1.3 h (range: 0.4–2.9 h), and the second phase 0.18 ± 0.06 %IA/L (6%) with an effective half-life of 26 h (15–52 h) [41]. The ratio of the activity concentration in bone marrow aspirates over blood has been quantified in several patients over time and was found to be approximately 1 for [177Lu]Lu-DOTA-TATE (mean ratio of 0.88, not significantly different from 1) [172]. The expression of γ-H2AX in blood lymphocytes has been observed to be elevated up to 48 and 72 h after [177Lu]Lu-SSRT therapy [41, 42]. A linear correlation was obtained between γ-H2AX + 53BP1 expression and absorbed dose to blood within the first hours after administration [41]. A trend was also found between γ-H2AX expression and tumour absorbed dose [42].

The blood TAC for [177Lu]Lu-PSMA also follows a biphasic pattern, with reported effective half-lives of 0.16 ± 0.09 and 10.8 ± 2.5 h [21]. The expression of γ-H2AX and 53BP1 in blood lymphocytes was found to increase within the first hours and decreased at later times after [177Lu]Lu-PSMA therapy, and linear correlations with the absorbed dose to blood within the first hours and the absorbed dose rate at later time points (48 h and 96 h) were described [43]. Possibly the number of foci per absorbed dose rate is lower for [177Lu]Lu-PSMA than for [177Lu]Lu-SSRT [44].

#### Methodological aspects

The red-marrow absorbed dose can either be indirectly estimated from image-based methods, or from blood-based measurements [177]. The mean absorbed dose to the red marrow ${\overline{D} }_{\mathrm{RM}}$ has TIA contributions from activity in red marrow ${\tilde{A }}_{\mathrm{RM}}$, bone ${\tilde{A }}_{\mathrm{bone}}$, other organs with high-activity uptake ${\tilde{A }}_{\mathrm{h}}$, and the remainder of the body ${\tilde{A }}_{\mathrm{RoB}}$ according to

12 $${\overline{D} }_{\mathrm{RM}}={\tilde{A }}_{\mathrm{RM}}\cdot S\left(\mathrm{RM}\leftarrow \mathrm{RM}\right)+{\tilde{A }}_{\mathrm{bone}}\cdot S\left(\mathrm{RM}\leftarrow \mathrm{bone}\right)+\sum_{h}{\tilde{A }}_{\mathrm{h}}\cdot S\left(\mathrm{RM}\leftarrow \mathrm{h}\right)+{\tilde{A }}_{\mathrm{RoB}}\cdot S\left(\mathrm{RM}\leftarrow \mathrm{RoB}\right) ,$$

with their corresponding $S$-values. Activity in the red marrow consists of activity bound to red-marrow cells, and blood perfusing through marrow space and the extracellular fluid. Assuming the administered compound does not specifically bind to red marrow cells, then the activity and TIA concentrations can be derived from blood samples by the assumption that $\left[{\tilde{A }}_{\mathrm{RM}}\right]=\left[{\tilde{A }}_{\mathrm{BL}}\right]\cdot RMBLR$, with $RMBLR$ representing the activity concentration in red marrow over blood (BL). The blood-based method for calculating the self-absorbed dose for red marrow, i.e., the first term in Eq. 12, then follows from:

13 $$\overline{D }\left(\mathrm{RM}\leftarrow \mathrm{RM}\right)=\left[{\tilde{A }}_{\mathrm{BL}}\right]\cdot RMBLR\cdot {m}_{\mathrm{RM},\mathrm{ ref}}\cdot S\left(\mathrm{RM}\leftarrow \mathrm{RM}\right) ,$$

where ${m}_{\mathrm{RM},\mathrm{ ref}}$ is the red-marrow mass in the reference phantom for the $S$-value (Table 4). The factor $RMBLR$ is generally considered to be 1, both for [177Lu]Lu-SSRT and for [177Lu]Lu-PSMA. Image-based estimation of the red marrow TAC has been made from serial imaging by planar whole-body scans [37, 38], hybrid planar-SPECT/CT [39], or SPECT/CT [40]. With serial SPECT/CT, the activity concentration is often determined from VOIs over the lumbar vertebrae due to their relatively large volume and location away from high-uptake regions that may otherwise contribute with misplaced counts due to limited spatial resolution and scatter.

**Table 4:** Data for red marrow, including the mass and self-dose$S$-values for 177Lu, according to Olinda v 2.1 and IDAC-Dose 2.1

	Mass (g)		$\mathrm{S}(\mathrm{RM }\leftarrow \mathrm{RM })$ (mGy MBq^−1^ h^−1^)	
	Male	Female	Male	Female
Olinda v. 2.1	1170	0.0414	900	0.0537
IDAC-Dose 2.1	1394	0.0349	1064	0.0457

The absorbed dose to blood has been used to investigate correlations to the expression of biomarkers for DNA damage [41]. The mean absorbed dose to blood, ${\overline{D} }_{\mathrm{BL}}$, is then calculated by summation of the self-dose and the γ-ray cross dose from the total body (TB), according to

14 $${\overline{D} }_{\mathrm{BL}}=\left[{\tilde{A }}_{\mathrm{BL}}\right]\cdot S\left(\mathrm{BL}\leftarrow \mathrm{BL}\right)+{\tilde{A }}_{\mathrm{TB}}\cdot {S}_{\gamma }\left(\mathrm{BL}\leftarrow \mathrm{TB}\right) .$$

The $S$-value for 1 mL blood has been determined for 177Lu from the assumption of LED, giving $S\left(\mathrm{BL}\leftarrow \mathrm{BL}\right)$ = 85.3 $\mathrm{Gy}/(\mathrm{GBq h / mL})$, see Table 2 [41]. The unit for $\left[{\tilde{A }}_{\mathrm{BL}}\right]$ should thus be $\mathrm{GBq h}/\mathrm{mL}$. The $S$-value for total-body for γ-rays was obtained as ${S}_{\gamma }\left(\mathrm{BL}\leftarrow \mathrm{TB}\right)={S}_{\gamma }\left(\mathrm{TB}\leftarrow \mathrm{TB}\right)/{M}_{\mathrm{TB}}^{2/3}$, where $M$ is the body weight and ${S}_{\gamma }\left(\mathrm{TB}\leftarrow \mathrm{TB}\right)$ = 0.00185 $\mathrm{Gy}/(\mathrm{GBq h})$ for 177Lu [41, 45].

Specific uptake in the skeleton is of concern for patients with bone metastases, which is generally observed in end-stage prostate cancer [74]. In such situations, image-based dosimetry is required to calculate the red marrow absorbed dose distribution. Large volumes of skeletal lesions will also influence the red marrow distribution in marrow space, which may be considered for dosimetry for [177Lu]Lu-PSMA [175] and [177Lu]Lu-SSRT therapies [178]. Furthermore, any free lutetium ions in the injected drug will bind to the skeleton (60% of the activity) and be deposited in the liver (10%) [179], which can be prevented by the addition of DTPA before radiopharmaceutical administration [180].

$S$-values for 177Lu are based on the distribution of red marrow in an average population, following ICRP 89 [160]. Table 4 lists self-dose $S$-values for two data sets. The difference in these models mostly relates to the red-marrow mass: IDAC-Dose 2.1 is based on data from ICRP 133 [84], where the red marrow also contains blood (4% of the total blood volume of the reference phantom).

#### Recommendations for dosimetry

Measurement of the activity concentration in the blood remains the most common method for red-marrow dosimetry. Sampling time points should be chosen to capture both the early TAC peak and the slower washout phase. An example sampling schedule is directly after administration, 10 min, 30 min, 60 min, 90 min, 120 min, 360 min, 24 h, and one later time-point. Image-based estimation of the red-marrow TAC can also be made using sequential planar or SPECT/CT whole-body imaging [175, 178]. Typically, 2 or 3 time-points are acquired up to 48 h, and at least one later time point to follow the slower component. For SPECT/CT-based methods, it is advisable to avoid or adjust for spill-in of counts from regions with skeletal metastases [178].

### Dosimetry for the salivary, lacrimal, and pituitary glands

#### Summary of available dosimetry data

**Fig. 4:**
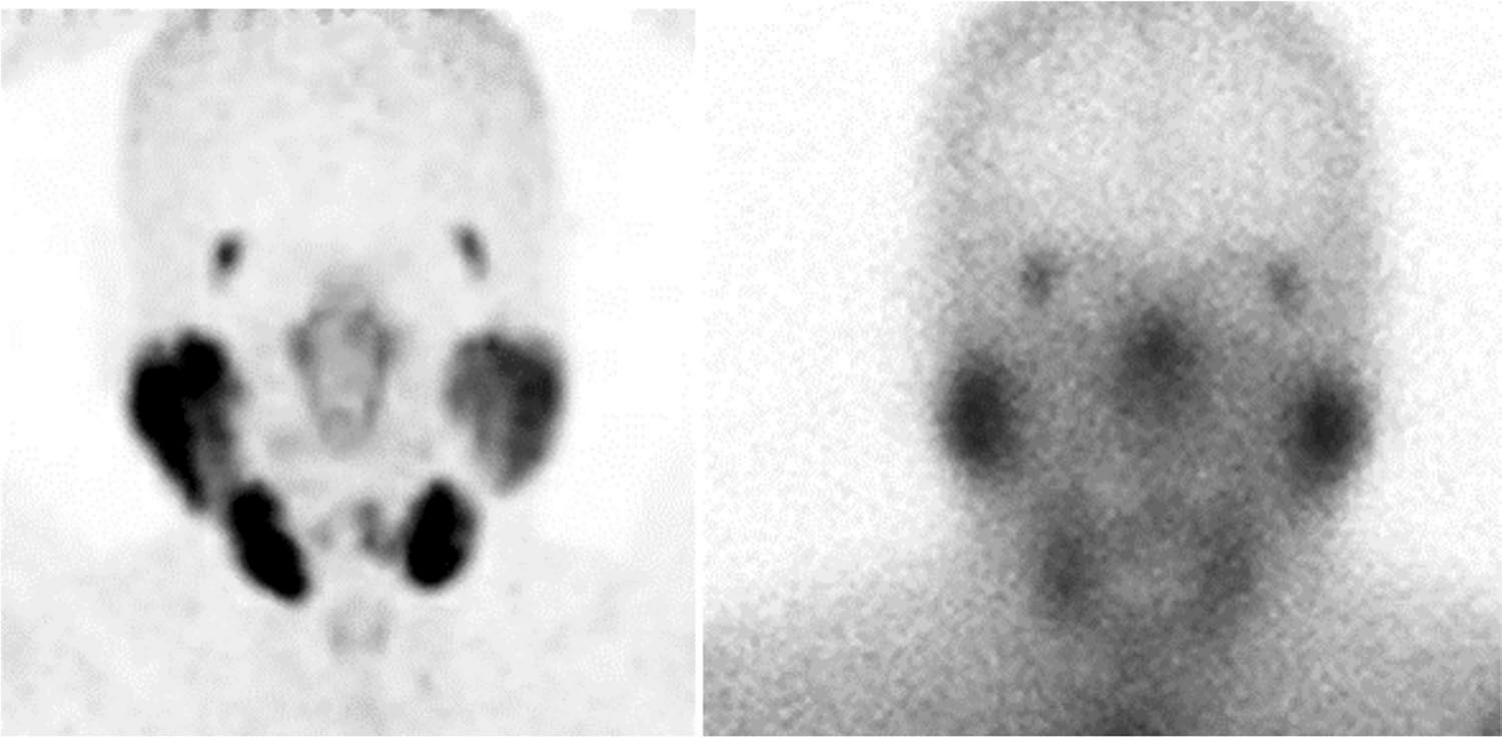
Anterior maximum-intensity projection of pre-therapy [68Ga]Ga-PSMA PET/CT (left) and [177Lu]Lu-PSMA-therapy gamma camera image (right) in a patient treated for metastatic prostate cancer. Large uptake can be observed in the different salivary glands and in lacrimal glands

Absorbed doses to salivary and lacrimal glands following [177Lu]Lu-PSMA therapy are summarised in Appendix 1, Table 6, and images acquired prior to, and during therapy with [177Lu]Lu-PSMA are shown in Fig. 4. As with other tissues, a large inter-patient variability is observed. For salivary glands, the absorbed doses range between 0.5 and 1.9 Gy/GBq. Studies that included dosimetry for more than one cycle demonstrated a modest variation between cycles [12, 23]. For lacrimal glands, the absorbed doses range between 0.4 and 3.8 Gy/GBq. One study reported a mean absorbed dose of 16 ± 4 Gy per therapy cycle, each of 5.5 GBq [177Lu]Lu-PSMA-617, which was almost 4 times higher than the absorbed dose to the salivary glands [67]. The lacrimal glands can potentially be considered the main organ at risk in therapy with 177Lu-PSMA, although presently, no significant concern of xerophthalmia has been reported [23].

**Fig. 5:**
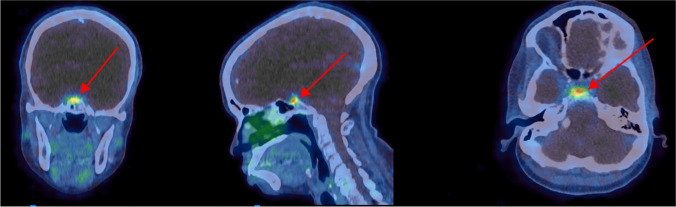
[68Ga]Ga-DOTA-TATE PET/CT of the head-and-neck region of a NET patient. Arrows indicate the radiopharmaceutical uptake in the pituitary region

The pituitary gland has a high expression of SSRs (Fig. 5). Absorbed doses to the pituitary gland following [177Lu]Lu-SSRT therapy have been investigated by planar image quantification with reported mean absorbed doses of 0.89 Gy/GBq (range 0.46–1.8 Gy/GBq) [69]. Radiobiological modelling was used to compare tolerance levels derived from EBRT, arriving at an EQD2 of 3.5 Gy (1.7–7.7 Gy) per 7.4 GBq cycle.

#### Methodological aspects

For the three pairs of salivary glands, the parotids have typical dimensions smaller than 5 cm in all directions, submandibular and sublingual glands have typical dimensions smaller than 4 cm and 2 cm, respectively [181]. The total mass of the three pairs of salivary glands is for the ICRP 110 reference adult male 85 g [182]. However, a large inter-patient variation in the salivary gland volume has been observed [183, 184].

Individual mass estimation of salivary glands for [177Lu]Lu-PSMA dosimetry is generally only made for the parotid and submandibular glands. Reported ranges are 31–43 g for both sets of glands [12]. Another study reported masses of 71 g for parotid and 28 g for submandibular glands [23]. The mass of lacrimal glands has been estimated to be approximately 1.4 g for both glands [67, 185]. The ICRP 89 pituitary gland mass is given as 0.6 g, while more recent volumetry has reported values of approximately 0.4 cm^3^ for individuals aged 50 years or more [186].

Activity quantification for salivary, lacrimal, and pituitary glands during [177Lu]Lu-PSMA or [177Lu]Lu-SSRT therapies was mostly based on planar imaging [12, 18, 23, 54, 67, 69, 176, 187, 188]. In the different imaging protocols employed, three to nine planar whole-body images were acquired between 0.5 and 192 h after administration. Activity quantification was carried out by direct delineation on planar images with or without background correction. Three studies of [177Lu]Lu-PSMA employed a hybrid imaging method (4 planar images up to 120 h + 1 SPECT/CT image at 24 h) [21] or only SPECT/CT images [22, 74]. No general trend could be observed when comparing absorbed doses calculated based on planar images only, a hybrid method, or SPECT/CT images only.

Although the use of planar-based activity quantification is recognised to suffer from the superposition of activity located in different tissues, for salivary and lacrimal glands the count contribution from overlapping tissues can be expected to be modest at later time points. However, at early time points (<3 h) activity in larger blood vessels may interfere with the activity estimate. Due to the small dimensions of these glands (<30 mL), the PVEs are large. In one study based on 3 SPECT/CT images, VOIs that included a 1- to 2-cm margin were applied to quantify activity in salivary and lacrimal glands [74]. Given the low concentration at later time points of [177Lu]Lu-PSMA in superimposed tissues, planar imaging can potentially yield sufficiently accurate activity estimates within the salivary and lacrimal glands. However, this would need confirmation by comparison to SPECT/CT. Planar-based activity quantification for the pituitary gland in [177Lu]Lu-SSRT therapy is hampered by possible activity uptake in the nasal mucosa, and SPECT/CT is recommended.

TACs of [177Lu]Lu-PSMA within the salivary and lacrimal glands exhibit an increase up to approximately 24 h post-administration and a constant rate of washout beyond this point [188]. Concerning the choice of the imaging time points, one study [67] compared the absorbed dose to salivary glands and lacrimal glands when using 4 imaging time points up to 72 h or when using an additional time point at 168 h. Results indicated that absorbed doses were overestimated when omitting the last time point by 20% for salivary glands and by 10% for lacrimal glands.

Most of the reported absorbed dose values have been calculated based on $S$-values for unit density spheres. For salivary glands, the $S$-value was adjusted to the patient-specific gland mass, measured using CT [18, 23], or was set to 85 g [67] corresponding to the mass given in ICRP 110 [182]. Lacrimal glands are not always easily delineable in CT images, although this technique was used with a mean value of 0.8 g in a cohort of 18 patients [23]. Accurate volumetry of the pituitary gland would likely require high-resolution MR imaging, and therefore individual mass estimation is challenging.

The ICRP 110 reference computer models include the salivary glands [182], and $S$-values for 177Lu are included in OpenDose [82], IDAC-Dose 2.1 [81], as well as Olinda v.2.1 (Table 5). For comparison, the $S$-value calculated based on the LED approach is also included in Table 5.

**Table 5:** Self-dose $S$-values for salivary, lacrimal, and pituitary glands, and unit density spheres, obtained from Olinda v.2.1, IDAC-Dose 2.1, and OpenDose. $S$-values for LED have been calculated based on ${\Delta }_{177\mathrm{Lu},\mathrm{e}}$ from ICRP 107 (Table 1) divided by the mass. Masses are retrieved from ICRP89 [160] except for lacrimal glands [67]. In their calculation process, IDAC-Dose 2.1 and OpenDose used slightly modified organ masses from ICRP 89, as indicated in brackets

	Mass (g) ICRP 89	$S$-value (mGy MBq^−1^ h^−1^)			Unit-density sphere $S$-value (mGy MBq^−1^ h^−1^)	
		Olinda 2.1	IDAC-Dose 2.1	OpenDose	IDAC-Dose 2.1	LED
Salivary glands (male)	85	1.00	0.947 (88.98 g)	0.994 (84.969 g)	1.01	1.00
Salivary glands (female)	70	1.22	1.17 (72.15 g)	1.20 (70.004 g)	1.23	1.22
Lacrimal glands	N/A	N/A	N/A	N/A	60.0 (1.4 g)	60.9 (1.4 g)
Pituitary gland (male)	0.6	N/A	127 (0.628 g)	133 (0.602 g)	137	142
Pituitary gland (female)	0.6	N/A	129 (0.618 g)	134 (0.597 g)	137	142

As noted, the differences between the values in Table 5 are modest and are probably mainly related to differences in the mass, the geometrical representation of these small regions, and possibly the radionuclide data used. For example, although IDAC-Dose 2.1 and OpenDose are based on the same computer phantoms from ICRP 110 and the same radionuclide data from ICRP 107, the $S$-values for salivary and pituitary glands differ, likely due to different voxel sizes that affect the mass used for calculation [82]. For absorbed dose calculation, any of these values may be used. When applicable, appropriate mass scaling should be applied according to the mass of the patient’s glands (Eq. 4).

Lacrimal glands are not included in the ICRP110 phantom, and precomputed $S$-values are not available. Therefore, $S$-values for unit density spheres or the LED approach must be used. In the absence of anatomical imaging, a mass of 1.4 g may be used, preferably also including a relevant range of masses to obtain an estimate of the standard uncertainty in the absorbed dose. The $S$-value for a unit density sphere with mass 1.4 g was used earlier [67], which corresponded to the average value of two other studies [185, 189].

The pituitary gland is included as a target region in OpenDose, which is thus the recommended $S$-value. The LED approach gives a value which is 6% higher than the $S$-value in OpenDose, probably due to the escape of electron energy for this small source region.

#### Recommendations for dosimetry

Dosimetry for salivary, lacrimal, and pituitary glands presents new and challenging tasks for physicists and physicians involved in [177Lu]Lu-SSRT and [177Lu]Lu-PSMA therapies. The challenge is mostly related to the small volume of these glands, which makes the patient-specific mass estimation difficult. For lacrimal and pituitary glands, dosimetry can be made assuming a standard mass that should preferably be varied across a realistic range to obtain an estimate of the uncertainty introduced by the mass assumption. Another challenge is the lack of studies that include sequential SPECT/CT over these glands. Thus, to date, there is no standardised approach for dosimetry for these glands. However, SPECT/CT-based dosimetry is encouraged as it is expected to provide important dosimetry data and a better understanding of the levels of the absorbed doses delivered.

### Dosimetry for tumours

#### Summary of available dosimetry data

Dosimetry results for tumours in [177Lu]Lu-SSRT therapy are summarised in Appendix 2, Table 7 [40, 72, 99, 171, 190–194]. For [177Lu]Lu-DOTA-TATE, a recent summary is also available [36]. A wide range of tumour-absorbed doses have been reported, between 0.1 and 32 Gy/GBq. For studies that included dosimetry in each cycle, it was noted that the tumour absorbed doses decreased between cycles [73, 99, 195]. One study observed a significantly more pronounced decrease for grade 2 than grade 1 NETs [195], while another observed a decrease for pancreatic but not small-intestinal NETs [73]. Effective half-lives were reported for a few studies and ranged between approximately 50 and 120 h, with shorter half-lives observed for grade 2 than for grade 1 NETs [171, 192, 195].

Table 8 summarises dosimetry studies for [177Lu]Lu-PSMA [12, 23, 74, 176, 187]. The mean values of reported tumour absorbed doses lie between approximately 1 and 8 Gy/GBq, with a trend of higher absorbed doses for bone metastases. The reported standard deviations have nearly equal magnitude as the mean, indicating a large variation between patients, tumours, and cycles. A trend of decreasing absorbed doses over cycles was observed [23]. In addition to individual-lesion dosimetry, SPECT/CT voxel dosimetry has been used to determine a whole-body tumour absorbed dose, calculated as the mean absorbed dose across all lesions receiving 5 Gy or more [74].

#### Methodological aspects

Whilst early studies used planar-based activity quantification of [177Lu]Lu-SSRT, there is a general transition towards sequential SPECT/CT or hybrid planar-SPECT/CT protocols. For [177Lu]Lu-PSMA, planar image-based activity quantification is still employed, possibly due to the large imaging FOV required to cover the entire extent of disease. For [177Lu]Lu-SSRT, a comparative study of different methods for activity quantification in the same cohort of patients reported tumour absorbed doses of 2.6 ± 1.5 Gy/GBq when using SPECT/CT only, 3.1 ± 2.2 Gy/GBq using a hybrid planar-SPECT/CT approach, and 5.3 ± 6.3 Gy/GBq using planar quantification [191]. Median and ranges were relatively comparable between SPECT/CT and hybrid planar-SPECT/CT but were considerably higher when only employing planar imaging. SPECT/CT-based activity quantification will enable standardisation, although different iterative reconstruction methods and their parameters may still yield a variable accuracy. For [177Lu]Lu-PSMA, using several FOV for the SPECT acquisitions is an attractive alternative [74], although performing sequential scanning for each cycle may be considered demanding in terms of patient comfort. Dosimetry methods based on simplified acquisition protocols are emerging [140]. Explicit recovery correction, using prior phantom imaging of spherical inserts for determination of the recovery coefficients, has been applied [12, 72, 192, 193, 195] and is a necessary requirement to obtain accurate tumour absorbed dose estimates from SPECT/CT.

Between three and five image acquisitions have generally been included, where the timing of the last acquisition has varied between 72 (3 d) and 168 h (7 d). To our knowledge, an explicit comparison of the impact of a late acquisition time has not been made for tumour dosimetry. However, the long biological half-life for tumours warrants a late time point. Most studies used $S$-values for unit density spheres, but the LED approach or voxel-based Monte Carlo were also used. The lesion mass was determined by delineation in diagnostic CT, SPECT/CT, or PET/CT.

#### Recommendations for dosimetry

Tumour dosimetry requires SPECT/CT for activity quantification, preferably using sequential SPECT/CT, or otherwise a hybrid planar-SPECT/CT approach. For the latter, only tumours that are not overlapped with other tissues with a pronounced activity accumulation can be included [192]. Application of explicit recovery correction is a pre-requisite, then taking the segmentation method and method for volumetry into account. The timing of image acquisitions is similar to those recommended for kidney dosimetry. The long biological half-time of the tumour retention emphasises the need for a late acquisition time point, see Figure 2.

## Discussion

Notable advances have been made in the field of radionuclide therapy with the introduction of new targeting molecules, radionuclides, equipment, technology, and methods for activity quantification and dosimetry. In parallel, theranostic approaches are expanding and the evidence of dose-effect correlations increasing [36, 74, 176, 196–198]. The radionuclide 177Lu has excellent characteristics for therapeutic imaging and a half-life that suits the pharmacokinetics of many radiotherapeutic compounds. These aspects offer advantages for theranostics and give the foundation for personalised, dosimetry-guided therapy based on [177Lu]Lu-SSRT and [177Lu]Lu-PSMA.

The absorbed dose tolerance of radiosensitive organs and the tumour absorbed doses required for treatment efficacy are not yet established. Organs at risk for therapy with [177Lu]Lu-SSRT are considered to be the kidneys, bone marrow, and possibly the pituitary gland. For [177Lu]Lu-PSMA therapy, the primary organs at risk are the parotid and lacrimal glands and bone marrow. The radiobiological reactions of the bone marrow and parotid glands are manifested both early and late, while kidneys and pituitary gland are generally regarded as late-responding tissues. Several studies have addressed dose-effect investigations for therapies with [177Lu]Lu-SSRT and [177Lu]Lu-PSMA. Relationships between tumour diameter or volume reduction and the absorbed dose evaluated at the time of best response were observed in [177Lu]Lu-SSRT therapy of NET [72, 73]. For [177Lu]Lu-PSMA, a significantly higher absorbed dose was observed for PSA-responders versus nonresponders when the mean absorbed dose was calculated across all metastases [74]. For [177Lu]Lu-SSRT therapy of NETs, dosimetry-guided trials have been undertaken with the aim of delivering a high tumour absorbed dose whilst respecting the absorbed dose or BED tolerance of the kidneys [32, 40, 55, 152, 199]. Modifications to the standard treatment protocol have included tailoring of the number of 7.4 GBq treatment cycles to the individual patient or modulation of the administered activity per cycle.

Large intra- and inter-patient variabilities in absorbed doses delivered during therapies with [177Lu]Lu-SSRT and [177Lu]Lu-PSMA have been demonstrated in several studies, both regarding tumours and normal organs [36, 140]. The observed variability may partly be the diversity of dosimetry methods and protocols applied at different centres, depending on experience, resources, and technology [135, 200, 201]. However, there is now an expanding interest in personalised dosimetry, and several initiatives have been taken to improve traceability in absorbed-dose estimates, uncertainty assessment, and consistency across centres [82, 102, 138, 202]. More profound reasons for the observed absorbed dose variability are the intrinsic characteristics of the patients, which govern the radiopharmaceutical uptake and washout for tumours and normal organs. As a consequence, the administration of the same amount of activity to all patients leads to a wide range of absorbed doses to tumours and critical organs. Given that the therapeutic effect is induced by ionising radiation, it is expected that personalization, including dosimetry, will lead to an improved risk-versus-benefit balance.

The practical implementation of dosimetry requires imaging at several time points after administration. For most of the tissues of dosimetry interest in therapy with [177Lu]Lu-SSRT and [177Lu]Lu-PSMA, we find that three acquisitions, well separated in time, are sufficient to capture the pharmacokinetics. Generally, the last image should be acquired at a time beyond the effective half-life for the particular tissue, and especially for tumours, this extends to many days after administration. For tissues where the typical pharmacokinetics is well known from previous patient cohorts, proposals have been made of using a lower number or even a single acquisition time point for dosimetry [139–141]. The use of pre-therapeutic 68Ga-PET/CT and its correlation to [177Lu]Lu-PSMA dosimetry of tumours and parotid glands have also been investigated [74]. Although recognising that such approaches of simplifying the dosimetry protocol need careful cross-validation, they offer advantages in terms of broadening the clinical use of dosimetry. In addition, pharmacokinetic modelling may assist in future dose planning [203, 204], as well as AI-based image segmentation methods [120].

Therapies with [177Lu]Lu-SSRT and [177Lu]Lu-PSMA are to be envisaged in an overall framework of precision medicine, involving imaging, clinical data, genetics, dosimetry, and radiobiology. As with other biomarkers, dosimetry data do not represent the only predictive parameter, but it is regarded as one among others that need to be taken into account. 177Lu is well suited for imaging and dosimetry-guided treatment schedules and can achieve the prerequisites for multicentre comparability. In addition, from a radiation protection point of view, there are legislative obligations of performing dosimetry for therapeutic nuclear medicine [205].

## Conclusions

There is a growing body of data on absorbed doses to organs and tumours in treatments with [177Lu]Lu-SSRT and [177Lu]Lu-PSMA. Together, such data provide an improved understanding of these therapies and may, in the long run, lead to the development of dosimetry-guided treatment protocols. The methods outlined in this report are not prescriptive but aim to harmonise data collection between centres in order to obtain comparable data. The methods should be within reach for all cancer centres that offer therapy with 177Lu-labelled compounds.

## Appendix 1

Table

**Table 6:** Summary of dosimetry data for salivary and lacrimal glands in [177Lu]Lu-PSMA treatment of metastatic castration-resistant prostate cancer

First author (year)	Ligand	# pats	Method for activity quantification	Method for AD calculation	AD per unit of administered activity (Gy/GBq) (Mean ± SD)	Mass (g) (mean ± SD) [range]	No. of considered cycles	Ref.
Delker (2016)	PSMA-617	5	Planar: 1 h, 24 h, 48 h, 72 h	Sphere S-values	Salivary: 1.4 ± 0.5	38 ± 5 [31–43]	2	[12]
Kabasakal (2015)	PSMA-617	7	Planar: 4 h, 24 h, 48 h, 120 h	Sphere S-values Mass from CT delineation	Parotid: 1.17 ± 0.31	-	1 (Pre-therapeutic tracer adm.)	[18]
Kratochwil (2016)	PSMA-617	4	Planar: 0.5 h, 3 h, 20 h, 44 h, 5−8 days	Sphere S-values Mass from PET/CT delineation	Parotid: 1.28 ± 0.40 Submandibular: 1.48 ± 0.37	-	2	[54]
Baum (2016)	PSMA I&T	30	Planar: 5 time-points 0.5 h to 118 h SPECT/CT: between 45 h and 118 h	Olinda v.1	Parotid: 1.3 ± 2.3, max: 9.5	-	1	[187]
Scarpa (2017)	PSMA-617	10	Hybrid (for abdomen): Planar: 0.5 h, 4 h, 24 h, 72 h, 96 h SPECT/CT: 24 h	Sphere S-values Mass from PET/CT delineation	Parotid: 0.56 ± 0.25, max: 1.040 Submandibular: 0.50 ± 0.15, max: 0.660 Lacrimal: 1.01 ± 0.69, max: 2.7	Volumes: Parotid: 24.98 mL, max: 34 mL Submandibular: 8.63 mL, max:10.35 mL	1	[206]
Hohberg (2016)	DKFZ-PSMA-617	9	Planar: 0.5 h, 24 h, 48 h, 72 h, 168 h	Olinda v.1 ( AFs for thyroid with mass adjustment ) For lacrimal: sphere S-values Fixed masses for salivary and lacrimal	Salivary (parotid + submandibular): 0.72 ± 0.14, max: 0.898 Lacrimal: 2.82 ± 0.76, max: 4.04	Salivary: 85 Lacrimal: 1.4	1	[67]
Fendler (2017)	PSMA-617	10	SPECT/CT: 24 h, 48 h, 72 h	No details	Salivary: 1.0 ± 0.6	-	2	[22]
Kabasakal (2017)	PSMA-617	6	Hybrid: Planar: 4 h, 24 h, 48 h, 120 h SPECT/CT: 24 h	Sphere S-values Mass from CT delineation	Parotid: 1.9 ± 1.19	-	1	[21]
Okamoto (2017)	PSMA I&T	18	Planar: 30–120 min, 24 h, 48 h, 72 h, 6–8 days	Sphere S-values Mass from PET/CT delineation	Parotid: 0.55 ± 0.14, max: 0.84 Submandibular: 0.64 ± 0.40, max: 1.70 Lacrimal: 3.8 ±1.4, max: 7.03	For one gland: Parotid: 19.1 ± 5.7 [8.0–35.6] Submandibular: 8.2 ± 1.9 [4.2–14.3] Lacrimal: 0.45 ± 0.12 [0.25–0.78]	From 1 to 4	[23]
Yadav (2017)	DKFZ-PSMA-617	26	Planar: 0.5 h, 3.5 h, 24 h, 48 h, 72 h, 96 h, 120 h, 144 h, 168 h	Sphere S-values Mass from SPECT/CT delineation	Parotid: 1.31 ± 0.22 Submandibular: 1.11 ± 0.39 Salivary glands (Parotid + Submandibular): 1.24 ± 0.27, max: 1.85	-	1	[188]
Violet (2019)	PSMA-617	30	SPECT/CT: 1 h, 24 h, 96 h	2 methods: Voxel-based, VDK Sphere S-values (mass from PET/CT delineation)	Parotid: 0.58 ± 0.43, max: 1.87 Submandibular: 0.44 ± 0.36, max: 1.75 Lacrimal: 0.36 ± 0.18, max: 0.81	-	1	[74]

AD, absorbed dose; VDK, voxel dose kernel

6

## Appendix 2

Tables

**Table 7:** Summary of dosimetry data for neurodendocrine tumours treated with [177Lu]Lu-DOTA-TATE, except where explicitly stated otherwise

First author (year)	# pats	Method for activity quantification	Effective half-life washout (h)	Method for AD calculation	AD per unit administered activity (Gy/GBq)	Ref.
Wehrmann (2007)	61	Planar: close to adm., 3 h, 20 h, 44 h, 68 h	Mean ± SD: 75.5 ± 20.9	Sphere S-value. Volume/mass from CT delineation	Mean ± SD: 9.7 ± 11	[171]
Wehrmann (2007) [177Lu]Lu-DOTA-NOC	8	As above	Mean ± SD: 66.7 ± 16.6	As above	Mean ± SD: 7.5 ± 8.3	
Garkavij (2010)	7	Hybrid: Planar: 1 h, 24 h, 96 h, 168 h SPECT/CT: 24 h or 96 h	-	LED, volume/mass from SPECT/CT delineation, mass from CT	Median (range): 6.7 (0.1–20)	[99]
Jackson (2013)	17	SPECT/CT: 4 h, 24 h, and 72 h	-	Voxel-based, DPK	AD per cycle: 21.4 ± 9.7 Gy (6.6–10.2 GBq per cycle)	[190]
Ilan (2015)	24	SPECT/CT: 24 h, 96 h, and 168 h	-	Sphere S-value, volume/mass from SPECT/CT delineation	Median (range): 6.8 (1.3–23) (for first cycle)	[72]
Kupitz (2017)	16	Planar and SPECT/CT: between 4 h and 168 h, all pats 4 h, 24 h, and 72 h	-	Sphere S-values. Volume/mass from CT delineation	Planar: mean ± SD: 5.3 ± 6.3 Median (range): 3.1 (0.18–25) Hybrid planar-SPECT/CT: mean ± SD: 3.1 ± 2.2 Median (range): 2.7 (0.20–7.9) SPECT/CT: mean ± SD: 2.6 ± 1.5 Median (range): 2.7 (0.16–5.4)	[191]
Roth (2018)	6	Planar and SPECT/CT: 1 h, 24 h, 96 h, 168 h	From SPECT/CT: Mean: 96 h Range: 84 h – 117 h	LED, volume from SPECT/CT delineation, mass from CT	Hybrid planar-SPECT/CT: mean: 3.6 SPECT/CT: mean: 3.9 Range: 1.3–7.3	[192]
Jahn (2019)	25	SPECT/CT: 24 h, 96 h, and 168 h	-	Sphere S-values. Volume/mass from SPECT/CT delineation	Mean: 4.7 Median (range): 3.8 (1.3–15.5)	[193]
Rudisile (2019)	35	SPECT/CT: 24 h, 48 h, and 72 h	-	Sphere S-values. Volume/mass from SPECT/CT delineation	Mean ± SD: 2.3 ± 1.83	[194]
Del Prete (2019)	34	SPECT/CT: 4 h, 24 h, and 72 h	-	Activity concentration, small VOI in high-uptake region. Sphere S-value	Median (range): 4.4 (0.1–32.0)	[40]
Jahn (2021)	48	SPECT/CT: 24 h, 96 h, and 168 h	-	Sphere S-values. Volume/mass from SPECT/CT delineation	Medians for cycles 1, 2, 3, 4, 5, $\geq$ 6 (retrieved from graph) Pancreatic (P): 6.6, 3.9, 2.8, 2.7, 2.6, 1.8 Small-intestine (SI): 4.6, 4.9, 4.1, 3.4, 4.1, 2.8 Range all cycles, grade P and SI: 0.7–23	[73]
Roth (2021)	41	Hybrid: Planar: 1 h, 24 h, 96 h, 168 h SPECT/CT: 24 h	Grade 1 mean: 103 h, CI: 96–109 h Grade 2 mean: 81 h, CI: 73–90 h	Voxel-based Monte Carlo, volume from SPECT/CT delineation, mass from CT	Medians for cycles 1, 2, 3, 4, 5, $\geq$ 6 Grade 1: 4.4, 4.4, 4.1, 3.5, 3.8, 3.2 Grade 2: 3.6, 3.1, 2, 1.6, 1.1, 0.8 Range all cycles, grade 1 and 2: 0.3–10	[195]

AD, absorbed dose; DPK, dose-point kernel; CI, confidence interval

7,

**Table 8:** Summary of dosimetry data for tumours in [177Lu]Lu-PSMA treatment of metastatic castration-resistant prostate cancer

First author (year)	Ligand	# pats	Method for activity quantification	Effective half-life washout (h)	Method for AD calculation	AD per unit administered activity (Gy/GBq) (mean ± SD)	Ref.
Delker (2016)	PSMA-617	5	SPECT/CT: 1 h, 24 h, 48 h, and 72 h	-	Sphere S-values. Mass from SPECT/CT delineation	Bone metastases: 5.3 ± 3.7 Lymph node metastases: 4.2 ± 5.3 Soft tissue metastases: 2.1 ± 0.8	[12]
Baum (2016)	PSMA I&T	30	Planar: 5 time points 0.5–118 h	Median (range): 51 (14–160)	Olinda v.1	Median (range): 3.3 (0.03–78)	[187]
Scarpa (2017)	PSMA-617	10	Planar: 0.5 h, 4 h, 24 h, 72 h, and 96 h	-	Sphere S-values. Mass from 68Ga-PET/CT delineation.	All: 2.8 ± 0.5 (range 1.1–7.2) Bone metastases: 3.40 ± 1.94 Lymph node metastases: 2.55 ± 0.42 Visceral lesions: 2.43 ± 0.78	[176]
Okamoto (2017)	PSMA I&T	18	Planar: 1 h, 24 h, 6–8 days, some pts 48 and 72 h	-	Sphere S-values. Mass from CT delineation.	All: 3.2 ± 2.6 (range 0.22–12) Bone metastases: 3.40 ± 2.7 Lymph node metastases: 3.2 ± 2.2 Liver metastases: 1.2 ± 0.67 Lung metastases: 1.75 ± 0.92	[23]
Violet (2019)	PSMA-617	30	SPECT/CT (2- or 3-bed-positions) 4 h, 24 h, and 96 h	-	Voxel-based, DPK. Mean AD across all lesions receiving > 5 Gy.	Bone metastases: 5.28 ± 2.46 Lymph node metastases: 3.91 ± 3.93	[74]

AD, absorbed dose

8

## Abbreviations

PSMA: Prostate-specific membrane antigen
[177Lu]Lu-PSMA: 177Lu-labelled small-molecule PSMA-targeting ligands
SSR: Somatostatin-receptor
[177Lu]Lu-SSRT: 177Lu-labelled somatostatin-receptor targeting ligands
BED: Biologically effective dose
EBRT: External-beam radiotherapy
TIA: Time-integrated activity
TAC: Time-activity curve
TEW: Triple-energy window
FOV: Field of view
ROI: Region of interest
VOI: Volume of interest
PVE: Partial-volume effect
AF: Absorbed fraction
